# Structural diversity-guided optimization of carbazole derivatives as potential cytotoxic agents

**DOI:** 10.3389/fchem.2023.1104868

**Published:** 2023-01-18

**Authors:** Zilin Gao, Yu Chen, Yufei Nie, Keming Chen, Xiufang Cao, Shaoyong Ke

**Affiliations:** ^1^ College of Science, Huazhong Agricultural University, Wuhan, China; ^2^ National Biopesticide Engineering Research Centre, Hubei Biopesticide Engineering Research Centre, Hubei Academy of Agricultural Sciences, Wuhan, China

**Keywords:** carbazole derivatives, synthesis, biological evaluation, anticancer, SARs

## Abstract

Carbazole alkaloids, as an important class of natural products, have been widely reported to have extensive biological activities. Based on our previous three-component reaction to construct carbazole scaffolds, we introduced a methylene group to provide a rotatable bond, and designed series of carbazole derivatives with structural diversity including carbazole amide, carbazole hydrazide and carbazole hydrazone. All synthesized carbazole derivatives were evaluated for their *in vitro* cytotoxic activity against 7901 (gastric adenocarcinoma), A875 (human melanoma) and MARC145 (African green monkey kidney) cell lines. The preliminary results indicated that compound **14a** exhibited high inhibitory activities on 7901 and A875 cancer cells with the lowest IC_50_ of 11.8 ± 1.26 and 9.77 ± 8.32 μM, respectively, which might be the new lead compound for discovery of novel carbazole-type anticancer agents.

## 1 Introduction

Cancer has long been an important disease threatening human health. Except the direct invasive therapy, radiotherapy, the use of chemotherapeutic drugs to kill cancer cells and treat tumors are the most important treatment of intermediate and advanced tumors with clinical metastasis (Sung et al., 2021). In the current clinical trials of cancer treatment, 65% of drugs are derived from natural products (Newman and Cragg, 2020; Bhutani et al., 2021). Despite the extensive use of drug design, compounds in nature are still the focus of drug research and development.

Natural products, especially heterocyclic compounds, have always been an important source of anticancer drugs and a molecular library of potential drugs (Fang et al., 2021; Yang et al., 2021). Many natural products or their derivatives have been demonstrated to have extensive antitumor activities by inducing apoptosis (Chen et al., 2015; Liu et al., 2015; Padmaja et al., 2015; Bailly, 2020; Greco et al., 2021) and inhibiting mitosis (Thomas et al., 2016; Davison and Sperry, 2017; Yokoyama et al., 2019). As an important class of aromatic heterocyclic compounds, carbazole alkaloid derivatives have long been used in medicinal chemistry (Murali et al., 2017; Sun et al., 2017; Zhou et al., 2018; Cao et al., 2019; Issa et al., 2019; Liu et al., 2019; Rassias et al., 2019; Liu et al., 2020; Liu et al., 2021; Wang et al., 2022), agrochemicals (Kong et al., 2016; Wang et al., 2017; Dang et al., 2018; Sun et al., 2020; Xie et al., 2020) and other fields. So, how to realize the functional modification of carbazole ring to improve its activity is always an important research direction.

**FIGURE 1:**
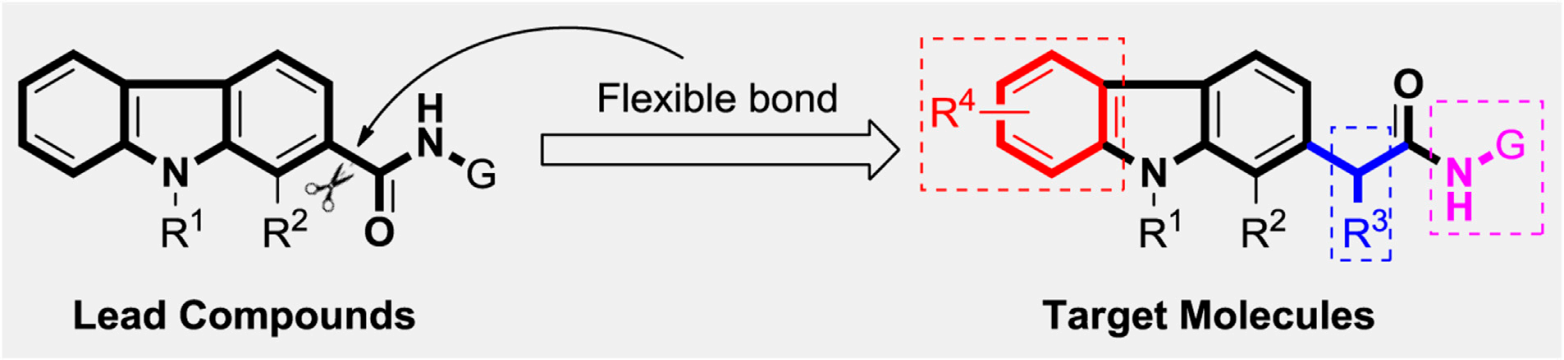
Design of novel carbazole alkaloid derivatives.

In our previous work, a series of thirty carbazole arylhydrazone derivatives were conveniently synthesized and evaluated as potential cytotoxic agents, using two of the same cells as this paper, namely A875 (human melanoma) and MARC145 (African green monkey kidney cell line MA-104) cells. Among them, compound **2021-7e** (Figure 5) elicited the lowest IC_50_ against A875 (4.15 μM) and IC_50_ of 26.64 μM on MARC145 (Huang et al., 2021). Meanwhile, a three-components indole-to-carbazole reaction (Wu et al., 2021) was adopted to construct the carbazole scaffold, which has high yield compared to the traditional reactions (Aggarwal and Verma, 2019; Wu et al., 2021). However, the carbazole unit is directly connected with the carbonyl group forming a p-π rigid conjugated system, which may not be conducive to the binding with the receptor protein. So, in order to further investigate the derivatization ability and drug potential of carbazole analogues, a methylene unit was introduced to provide a rotatable bond attached to the carbazole scaffold (Figure 1), and a series of carbazole derivatives with structural diversity including carbazole amide, carbazole hydrazide and carbazole hydrazone were designed and synthesized. We hope that the introduction of this flexibility is proposed to increase the binding ability of target molecules to receptor proteins.

**SCHEME 1:**
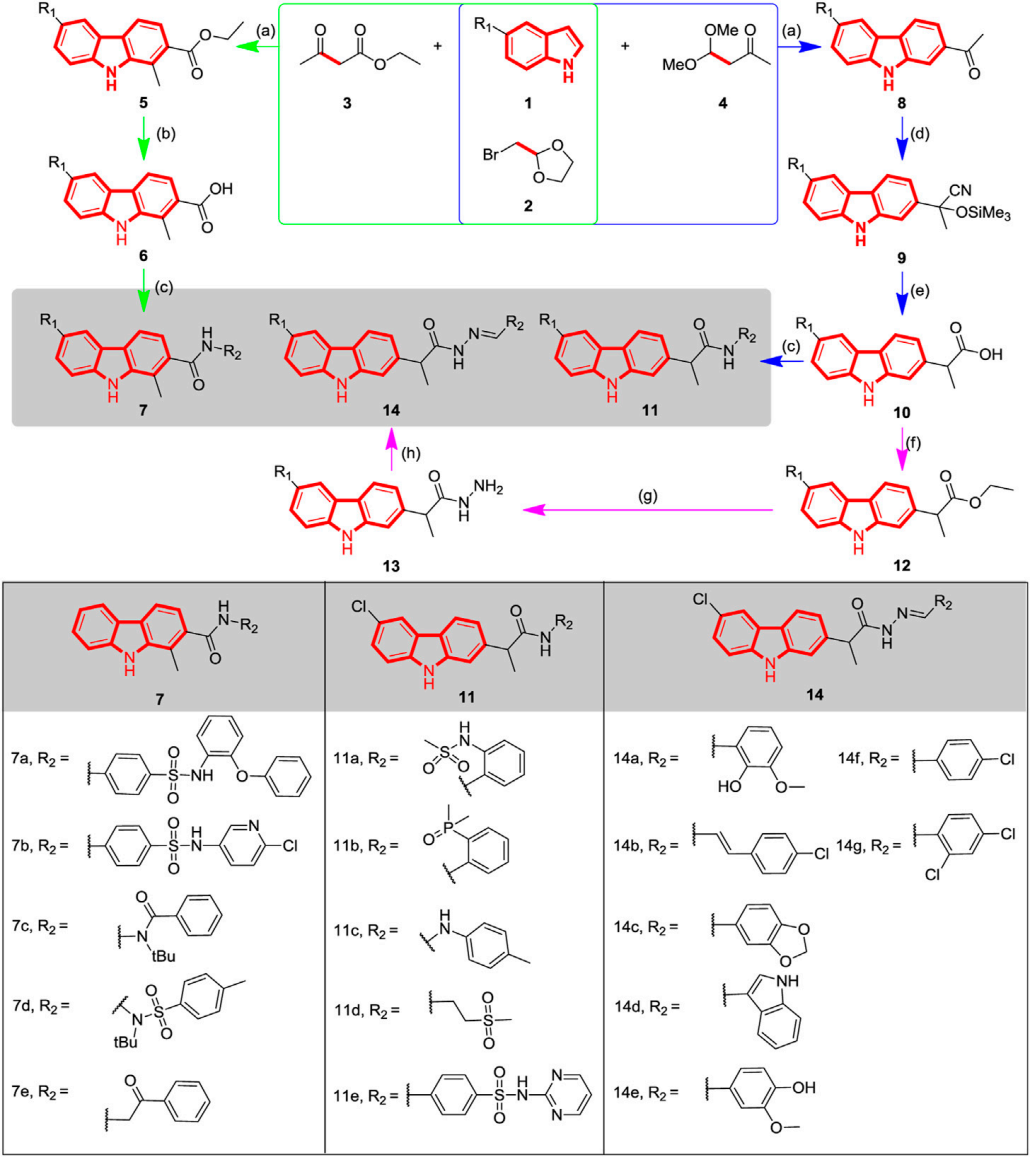
Synthetic route and structure of carbazole derivatives **7a-e**, **11a-e** and **14a-g**. Reagents and conditions: (a) AlCl_3_, CH_3_CN, reflux; (b) KOH, EtOH, rt; (c) i. Py, MsCl, CH_3_CN, 0°C; ii. R_2_-NH_2_ or R_2_=NNH_2_, 40-50 °C; (d) Me_3_SiCN, ZnI_2_, rt; (e) i. SnCl_2_, AcOH, rt; ii. conc. HCl; (f) EtOH, Conc. H_2_SO_4_, reflux; (g) NH_2_NH_2_·H_2_O, EtOH, reflux; (h) Aldehyde, EtOH, reflux.

Thus, we wish to report herein the convenient synthesis of novel carbazole derivatives with structural diversity as indicated in Scheme 1, and their potential cytotoxic activities against several cell lines including human gastric adenocarcinoma cells (7901), human melanoma (A875), and a subclone of African green monkey kidney cell line MA-104 (MARC145) were investigated using classical MTT colorimetric method.

## 2 Results and discussion

### 2.1 Chemistry

In this work, three series of carbazole analogues with structural diversity were designed and synthesized by integrating the carbazole alkaloid moiety with diverse pharmacophores including amide, hydrazide and acylhydrazone *etc.* The general synthetic route and structures is depicted in Scheme 1.

In previous studies it was demonstrated that a series of carbazole structures could be obtained by the three-component reaction of bromoacetal acetal, ketone and indole catalyzed by bismuth trifluoromethanesulfonate (Gu et al., 2018), but the carbazole structure is directly connected to the carbonyl group, which formed a rigid conjugated structure. In order to further explore the potential effect of adding rotatable bonds to improve molecular flexibility on activity, different types of carbazole derivatives were designed and synthesized. Firstly, 1-methyl-9*H*-carbazoles **7a-e** were synthesized via three steps according to our previous procedure. The carbazole scaffold **5** was synthesized using indole, ethyl acetoacetate and 2-(bromomethyl)-1,3-dioxolane, which was then hydrolyzed to obtain the corresponding acids **6**, and then treated with a pharmacophore-bearing amine to form various amide derivatives. Similarly, the key carbazole acids with rotatable bonds of type **10** were constructed via three steps (Percy et al., 1994) as indicated in Scheme 1; In particular, the carbazole acetyl **8** was obtained using a similar method to compounds **5** by using ethyl acetoacetate instead of 4,4-dimethoxybutan-2-one. Therefore, the carbonyl group of compounds **8** was cyanated through trimethylcyanosilane, and then hydrolyzed to obtain the intermediate carbazole acids **10**, which were isolated as a white solid by column chromatography. A series of compounds **11a-e** were conveniently synthesized by direct reaction of carbazole acids **10** with the appropriate amines. Compounds **10** were also transformed into the corresponding carbazole hydrazide **13** via two steps: esterification of the acid group followed by reaction with hydrazine hydrate. The compounds **13** were also reacted with a carbonyl-bearing substrate to obtain compounds **14a-g**, which bridges the carbazole structure to the potentially active pharmacophore (Popiolek et al., 2018; Xie et al., 2019; Duangdee et al., 2020; Han et al., 2020; Peng et al., 2020; Wang et al., 2020). Because the condensation method used is not stereoselective, the resulting acylhydrazone derivatives are a mixed (E/Z) configuration.

### 2.2 Spectroscopy

**FIGURE 2:**
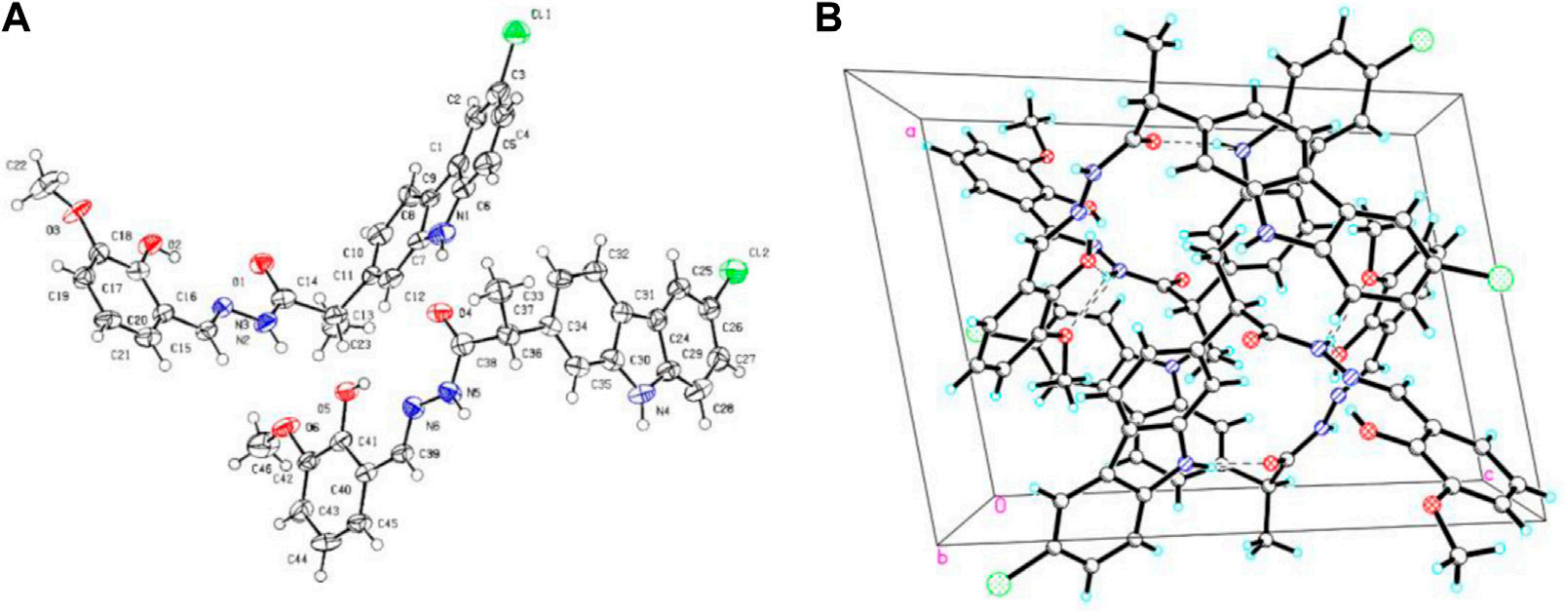
Single crystal structure **(A)** and packing diagram **(B)** for compound **14a**, and the dashed lines denote intermolecular hydrogen bonds.

All obtained carbazole derivatives were elucidated based on the chemical analyses including ^1^H NMR, ^1^C NMR, ESI-MS, HRMS, and X-Ray analyses. For the ^1^H NMR spectra of all 1-methyl-9*H*-carbazole derivatives **7a-e**, the obvious signals at 2.08–2.66 ppm were attributed to the methyl group attached to the carbazole ring as shown in the molecular structure. With respect to other carbazole derivatives **11a-e** and **14a-g**, the ^1^H NMR spectrum indicated a unique signal for the flexible bond CHCH_3_ unit, with a quartet signal at about 4.92–3.73 ppm and a doublet signal at about 1.63–1.40 ppm, respectively. Both the NH protons on the carbazole ring and the NH protons next to the carbonyl appear in the range of 11.52–9.56 ppm. The remaining signal peaks at low fields are attributed to the proton peaks of the carbazole ring or other aromatic rings. All the characteristic peaks observed within the ^1^H NMR spectra for title compounds are given in materials and methods. In addition, the representative single crystal diffraction analysis of target compound **14a** is shown in Figure 2.

### 2.3 Pharmacology evaluation

**FIGURE 3:**
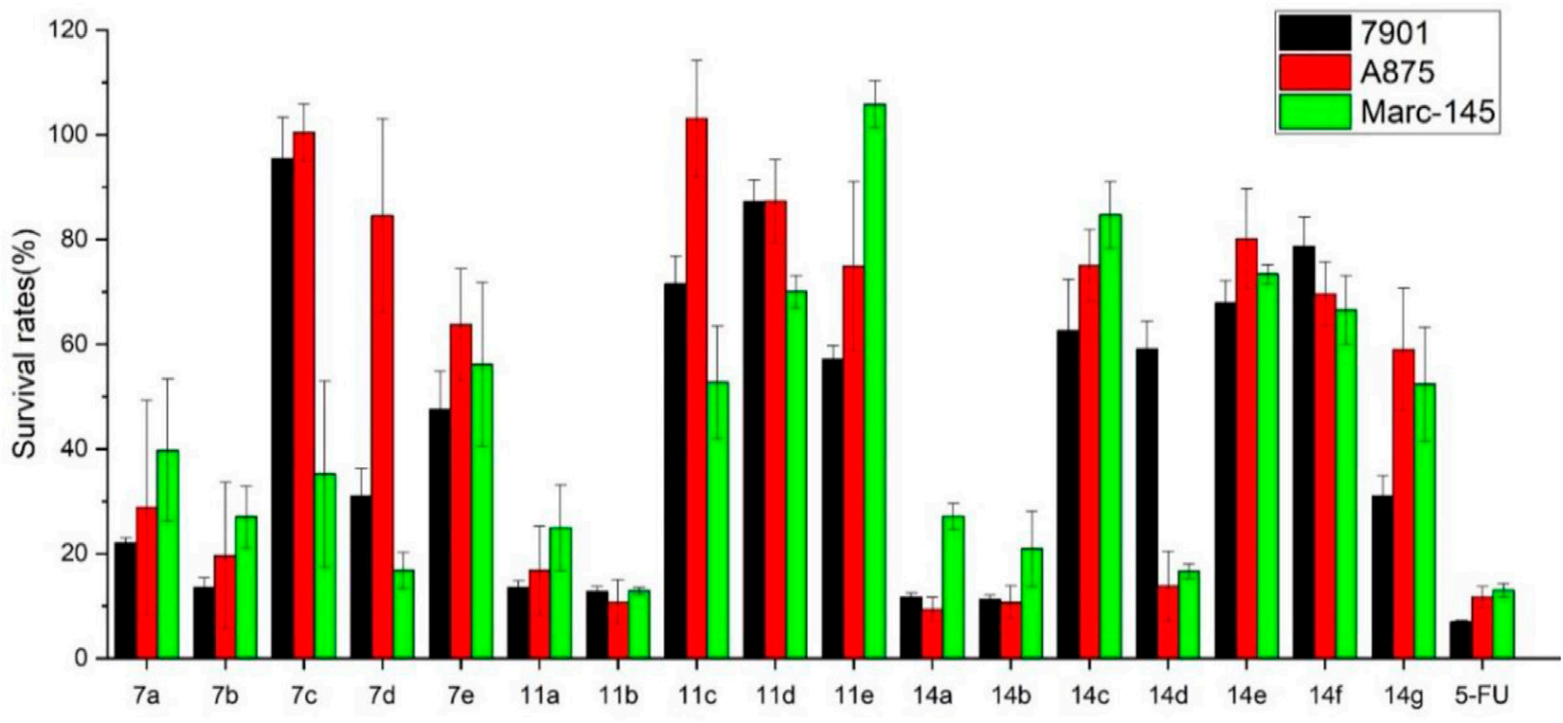
Cytotoxic activities of synthesized compounds at the concentration of 40 μg/mL. Abbreviations: 7901—Human gastric cancer cell line; A875—Human melanoma cell line; MARC145—A subclone of African green monkey kidney cell line MA-104; 5-FU—5-Fluorouracil, used as a positive control.

Using 5-fluorouracil (5-FU) as a positive control, the inhibition effects of all carbazole derivatives on 7901 (gastric adenocarcinoma cells), A875 (human melanoma) and MARC145 (African green monkey kidney cell line MA-104) cells *in vitro* were evaluated by standard MTT assay, and the preliminary bioassay results were described in Figure 3. Generally, as shown in Figure 3, some compounds showed higher activities compared with the control 5-FU. Among them, carbazole derivatives **7b**, **11a**, **11b**, **14a**, and **14b** had higher *in vitro* inhibition rates on the two cancer cells 7901 and A875 (>70%). In addition, compounds **7b**, **11a** and **14a** had less toxicity to normal cells MARC-145 than cancer cells, especially, it is worth noting that, compound **14a** have little toxicity to normal cells MARC-145 and show moderate selectivity to cancer cells.

Through the preliminary screening results, some molecules showed good inhibitory effects on target cancer cells. In order to further verify the potential activity of these carbazole derivatives, we determined the IC_50_ values of all compounds **7a-e**, **11a-e**, and **14a-g**. It is further proved that compounds **11a**, **11b**, **14a**, **14b** are the highly potential scaffolds with excellent anticancer activities.

Generally, the carbazole derivatives with flexible bonds attached to carbazole ring have some advantages compared with the carbazole derivatives with rigid structures, and overall exhibit better inhibitory activity against cancer cells. It is worth noting that compounds **11a**, **11b**, **14a**, and **14b** exhibited good inhibition activities on all tested cells. Especially, compound **14a** derived from *o*-vanillin was most active molecule against all tested cell lines with the IC_50_ value of 11.8, 9.77, and 7.47 μM, respectively, which was significantly better than that of the control 5-FU. However, compound **14e** containing vanillin scaffold almost lost its activity, which indicated the hydrophilic groups in the neighborhood of the aromatic ring might be very important for the activity of these class of compounds. In addition, the four compounds **11a**, **11b**, **14a**, **14b** have similar structures to carprofen and may act as a pharmacophore to enhance its antitumor activity (Favia et al., 2012; Mellini et al., 2012; Zhou et al., 2018).

**FIGURE 4:**
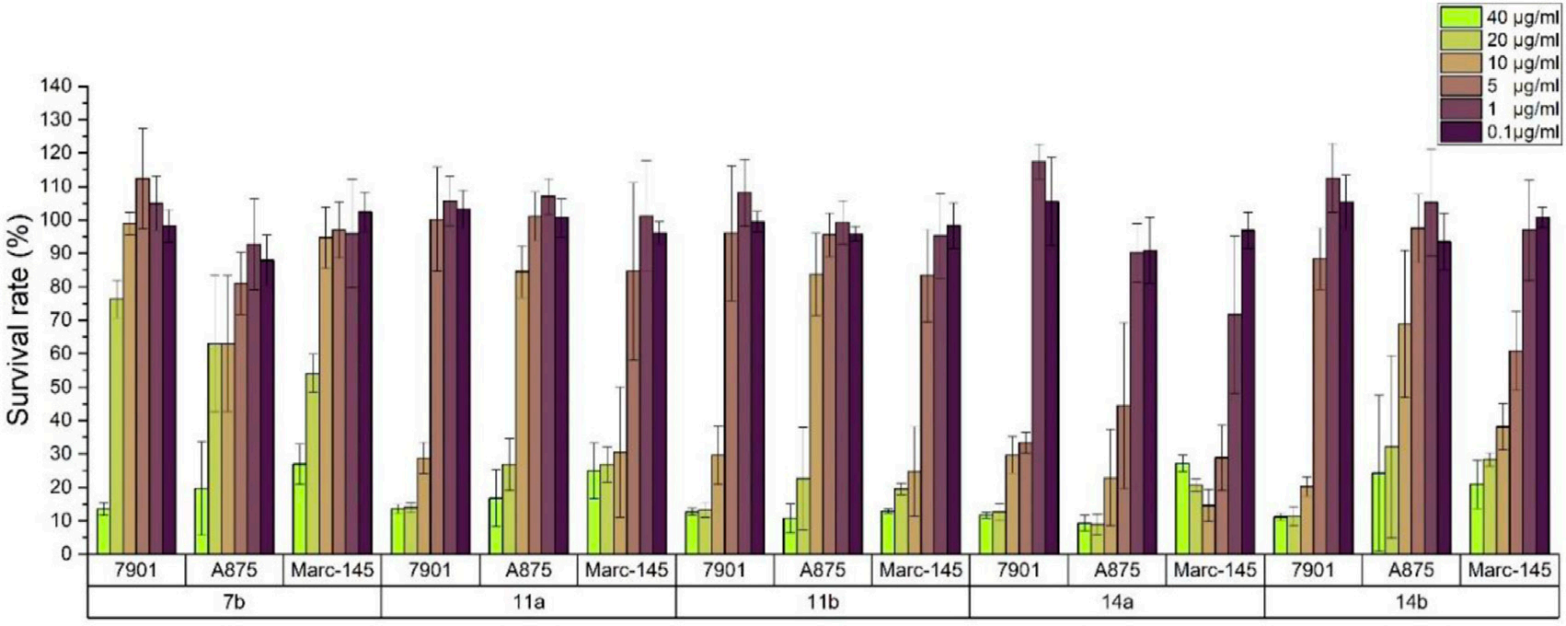
Survival rates of 7901, A875 and Marc-145 in different concentration of **7b**,**11a**, **11b**, and **14a**, **14b**.

The relationship between concentration and inhibition rate is the key to whether a potential compound can be use as potential medicinal agents. It is very important to maintain good activity at low concentration. Based on the preliminary screening data, in order to verify the effect of concentration on the potential cytotoxic activities for all compounds, the inhibition activity data of the compounds at different concentrations were detected using the same method. All experiments were carried out on three kinds of cell lines with six concentrations of 40, 20, 10, 5, 1, and 0.5 μg/mL. The inhibition activities for highly potential compounds 7b, 11a, 11b, 14a, and 14b were described in Figure 4. It can be seen that with the decrease of the concentration, the inhibition rate of the cells also decreased, but good activity was also guaranteed for compound **14a** at a concentration of 5 μg/mL, which was significantly better than that of other compounds tested.

**FIGURE 5:**
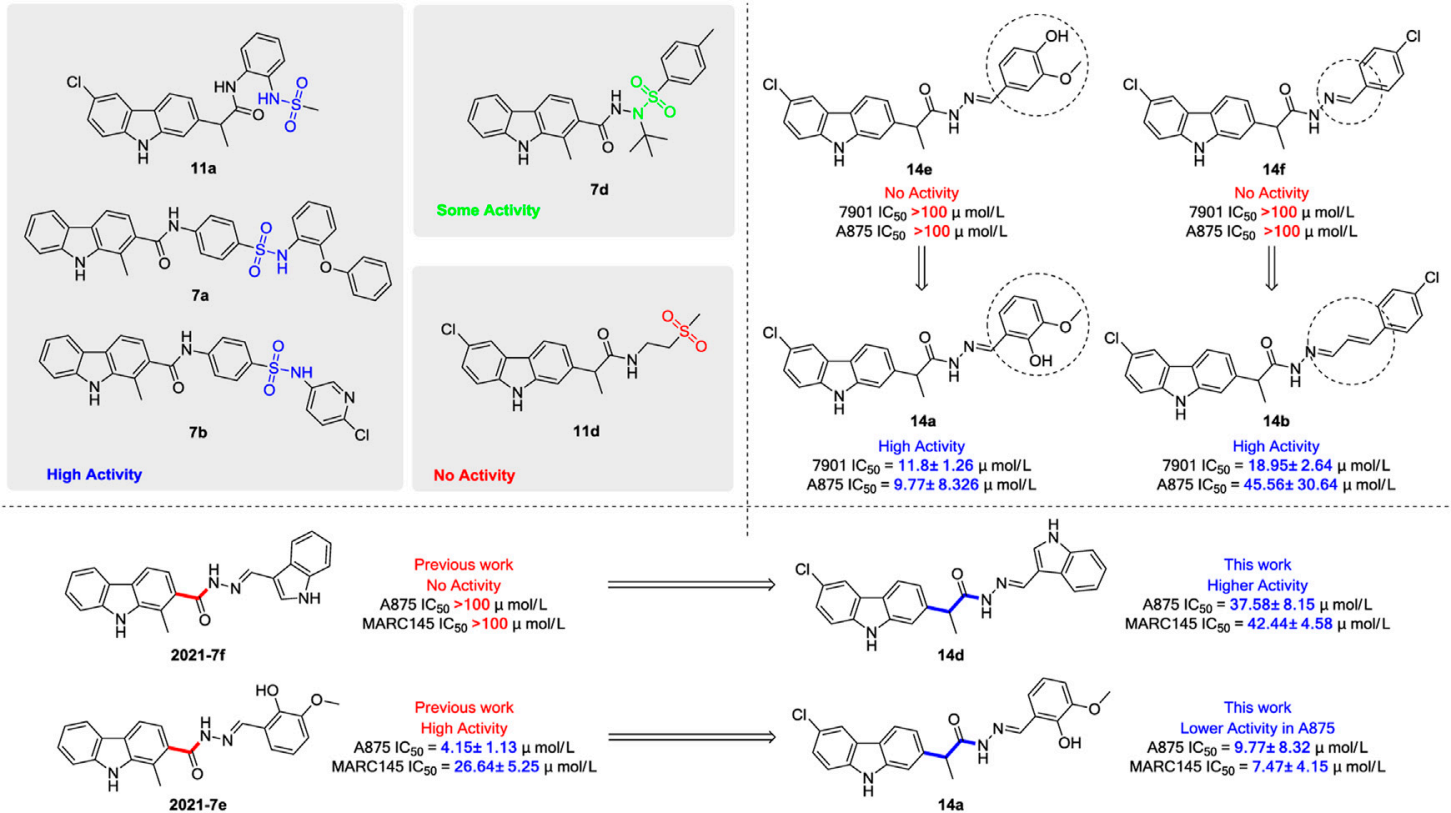
General structure-activity profile for these carbazole derivatives and previous work.

The structure evolution here was to modify carbazole scaffold and different pharmacophore. According to the *in vitro* results described in Table 1, the possible structure-activity relationships for these synthesized carbazole derivatives can be concluded. Considering the type of pharmacophore, it can be clearly seen that the sulfonamide group can efficiently improve the potential activities of these compounds as indicated in Figure 5. When there is no hydrogen on the amino group, the compound’s activity (such as 7d) decreases significantly, as it may affect the formation of hydrogen bonds. However, the molecule **11d** containing only sulfone group also lost activity. The structural isomerism and the length of the carbon chain significantly affected the activity of the compound. For example, the activity of compound **14a** bearing 2-hydroxy-3-methoxybenzylidene unit is significantly better than that of compound **14e** containing 4-hydroxy-3-methoxybenzylidene moiety, as well as for compound **14b** characterized with one more vinyl group than derivative **14f**. In contrast to the previous work, the addition of flexible bonds produced complex effects. On the one hand, the activity of some compounds was improved (such as **14d**), likely due to the increase of rotatable bonds which i) reduced the permeability to the cell membrane, or ii) increased the entropy penalty when binding proteins. However, the rotatable bonds also reduced the activity of some compounds (for example, compounds **14a**). It is worth noting that the 2-hydroxy-3-methoxybenzylidene group is the most effective pharmacophore in the previous work (Compound **2021-7e**) and this work (Compound **14a**), and has the value of further research (Huang et al., 2021) (Figure 5).

**TABLE 1:** *In vitro* cytotoxic activities of target compounds against tested cell lines.

Entry	Compd. No.	Substituents		*In vitro* cytotoxicity IC_50_ ^a^ (μM)		
		R_1_	R_2_	7901^*b*^	A875*^b^*	MARC145*^b^*
**1**	**7a**	H	, 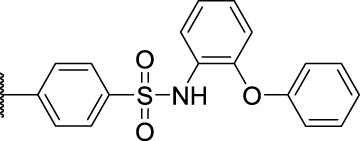	64.72 ± 15.12	33.53 ± 1.63	51.49 ± 9.24
2	**7b**	H	, 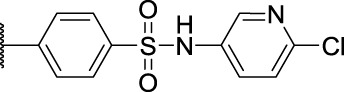	58.11 ± 11.26	23.04 ± 16.95	58.88 ± 16.68
3	**7c**	H	, 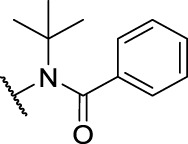	>40	>40	58.4 ± 31.59
4	**7d**	H	, 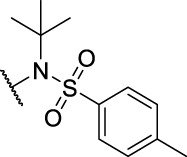	34.52 ± 9.9	>40	18.73 ± 4.16
5	**7e**	H	, 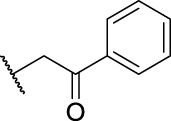	>40	>40	62.59 ± 28.21
6	**11a**	Cl	, 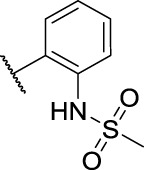	21.9 ± 3.05	36.68 ± 2.96	22.94 ± 14.3
7	**11b**	Cl	, 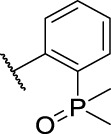	20.57 ± 3.3	33.62 ± 9.48	17.32 ± 7.25
8	**11c**	Cl	, 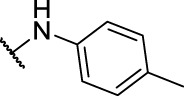	>40	>40	63.96 ± 20.11
9	**11d**	Cl	, 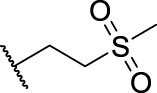	>40	>40	>40
10	**11e**	Cl	, 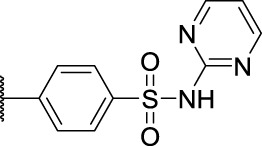	>40	>40	>40
11	**14a**	Cl	, 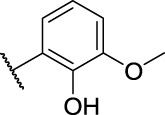	11.8 ± 1.26	9.77 ± 8.32	7.47 ± 4.15
12	**14b**	Cl	, 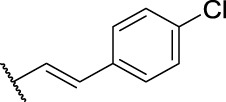	18.95 ± 2.64	45.56 ± 30.64	19.57 ± 6.94
13	**14c**	Cl	, 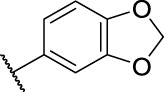	>40	>40	>40
14	**14d**	Cl	, 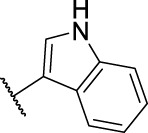	>40	37.58 ± 8.15	42.44 ± 4.58
15	**14e**	Cl	, 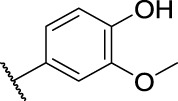	>40	>40	>40
16	**14f**	Cl	, 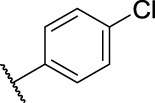	>40	>40	>40
17	**14g**	Cl	, 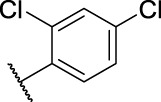	38.83 ± 2.74	>40	96.26 ± 63.3
18	**5-FU** *^c^*	-	-	63.5 ± 4.92	80.11 ± 6.61	86.33 ± 8.3

^a^ IC_50_, Compound concentration required to inhibit tumor cell proliferation by 50%.

^b^ Abbreviations: 7901, Human gastric carcinoma cell line; A875, Human melanoma cell line; MARC145, A subclone of African green monkey kidney cell line MA-104.

^c^ Used as a positive control.

According to the aforementioned analysis, the potential inhibitor activity is relatively good for those carbazole derivatives with higher molecular flexibility. In the rigid structure, only **7b** has moderate inhibitory activity on tested cells, which reflects the effect of molecular flexibility on the activity of these compounds. The increase of rotatable bonds may facilitate the binding of compounds to possible receptors, so as to improve its inhibitory activity (Peng et al., 2019; Caron et al., 2020).

## 3 Conclusion

In this study, several types of carbazole derivatives including carbazole amides, carbazole hydrazides and carbazole hydrazones were designed and synthesized based on the scaffold of carbazole alkaloids. All obtained molecules were fully characterized by ^1^H NMR, ^13^C NMR, ESI-MS, HRMS and X-Ray diffraction analyses, and their potential inhibitory activities on cancer cell lines were bio-evaluated. The bioassay indicated that some of the target compounds had good inhibitory effects on 7901 and A875 cancer cells, especially, the best potential compound **14a** exhibited high inhibitory activities with the lowest IC_50_ of 11.8 ± 1.26 and 9.77 ± 8.32 μM, respectively, which might be the new lead molecule for discovery of novel carbazole-type anticancer agents.

## 4 Materials and methods

### 4.1 Instrumentation and chemicals

All melting points (m.p.) were measured using a digital model X-5 apparatus and are uncorrected. ^1^H NMR and ^13^C NMR spectra were recorded on a Bruker Avance III 600 MHz FT-NMR spectrometer using DMSO-*d*_*6*_ as the solvent and tetramethylsilane (TMS) as the internal standard. Mass spectra were performed on a WATERS ACQUITY UPLC^®^ H-CLASS PDA (Waters^®^) instrument. High resolution mass spectrometry analysis (HRMS) was done with Agilent 6224 TOF mass spectrometer. Thin-layer chromatography was carried out on precoated silica gel plates GF254 (Qingdao Haiyang Chemical, China), and spots were visualized with ultraviolet light. All starting materials and reagents commercially available were used without further purification, unless otherwise specified.

### 4.2 General synthetic procedure for 1-methyl-9H-carbazole-2-carboxylic acid **6**

Indole (10.0 mmol), ethyl acetoacetate (10.0 mmol), 2-(bromomethyl)-1,3-dioxolane (12.0 mmol), AlCl_3_ (0.5 mmol, 5 mol%) and acetonitrile (35 mL) was added in a 100 mL single-neck flask. The mixture was stirred at 80°C for 6 h obtaining a solid which was filtered under reduced pressure. The residue product was rinsed by brine, and the aqueous phase was extracted by ethyl acetate. The organic phase was combined, dried over anhydrous Na_2_SO_4_ and filtered. The solvent was removed by rotary evaporation to obtain the crude intermediate **5**. The latter was added in a 100 mL single-neck flask with methanol (10 mL) and sodium hydroxide (15 mmol), and the reaction was stirred at room temperature overnight. The mixture was added dilute hydrochloric acid adjust to pH 2-3 under ice-bath, and the precipitate was 1-methyl-9*H*-carbazole-2-carboxylic acid **6**. The compound was brown powder, MS (ESI) *m/z* calcd for C_14_H_10_NO_2_ [M - H]^-^ 224.08, found 224.48.

### 4.3 General synthetic procedure for 2-(6-chloro-9H-carbazol-2-yl)propanoic acid **10**

Indole (10.0 mmol), 4,4-dimethoxybutan-2-one (10.0 mmol), 2-(bromomethyl)-1,3-dioxolane (12.0 mmol), AlCl_3_ (0.5 mmol, 5 mol%) and acetonitrile (35 mL) was added in a 100 mL single-neck flask. The mixture was stirred at 80°C for 8 h. After reaction, the solid was filtered under reduced pressure. The residue product was rinsed by brine, and the aqueous phase was extracted by ethyl acetate, then combine the residue and the organic phase, which was dried over anhydrous Na_2_SO_4_. After that, the solvent was removed by rotary evaporation, and the residue was used directly in subsequent reactions without purification. The residue was added in a 100 mL single-neck flask, and treated with trimethylsilyl cyanide and ZnI_2_ under the condition of reflux. After reaction, the mixture was cooled to room temperature and filtered, which was concentrated to obtain crude product **9**. Subsequently, compounds **9** was treated with stannous chloride in acetic acid, then hydrolysis with concentrated hydrochloric acid, and the carbazole derivative 2-(6-chloro-9*H*-carbazol-2-yl)propanoic acid **10** will be obtained through a series of acid-base treatment. MS (ESI) *m/z* calcd for C_15_H_12_ClNO_2_ [M - H]^-^ 272.06, found 272.41.

### 4.4 General synthetic procedure for 2-(6-chloro-9H-carbazol-2-yl)propanehydrazide **13**

To a solution of substituted 2-(6-chloro-9*H*-carbazol-2-yl)propanoic acid **10** (10 mmol) in ethanol was added the catalytic amount of concentrated sulfuric acid, which was refluxed at 80°C for 6–7 h. Then, the solution was washed with water and extracted with ethyl acetate, and the organic phase was directly used for the next reaction after drying and concentration. The residue was dissolved in 10 mL of ethanol, and 5 eq. of hydrazine hydrate was added, the mixture was heated at 70–75°C for 4 h. The precipitate was filtered and washed with a small amount of cold ethanol to obtain the desired molecule 2-(6-chloro-9*H*-carbazol-2-yl)propanehydrazide **13**: MS (ESI) *m/z* calcd for C_15_H_14_ClN_3_O [M + H]^+^ 288.08, found 288.25.

### 4.5 General synthetic procedure for the target compounds **7a-e** and **11a-e**

The general synthetic procedure of carbazole-based amide **7a-e** and **11a-e**: To a solution of carbazole-based carboxylic acids **6** or **10** (0.8 mmol) in 10 mL anhydrous acetonitrile was added pyridine (2.4 mmol) under the condition of 0–5°C. Then, methanesulfonyl chloride (1.2 mmol) was added dropwise to the mixture, which was then allowed to warm to room temperature and stirred for additional half hour, the various amines (0.8 mmol) were added, and the reaction mixture was heated to 40–45°C and detected by thin-layer chromatography. After completion of the reaction, the mixture was quenched by the addition of water, and the suspended solid was collected by filtration and washed with water to afford the crude products, which were purified by silica gel column chromatography (petroleum ether/ethyl acetate) to give the target compounds. Their physico-chemical properties and the spectra data are as follows:

1-Methyl-*N*-(4-(*N*-(2-phenoxyphenyl)sulfamoyl)phenyl)-9*H*-carbazole-2-carboxamide (**7a**): white solid; yield: 71%; m. p. 78°C; ^1^H NMR (600 MHz, DMSO-*d*_6_, 25°C) *δ* = 11.42 (s, 1H), 10.69 (s, 1H), 9.87 (s, 1H), 8.17 (d, *J* = 7.8 Hz, 1H), 8.08 (d, *J* = 8.0 Hz, 1H), 7.87—7.82 (m, 2H), 7.71—7.66 (m, 2H), 7.56 (dt, *J* = 8.1, 0.9 Hz, 1H), 7.46—7.40 (m, 2H), 7.33—7.29 (m, 3H), 7.21—7.18 (m, 1H), 7.12—7.08 (m, 3H), 6.75—6.71 (m, 1H), 6.71—6.67 (m, 2H), 2.65 (s, 3H) ppm; ^13^C NMR (151 MHz, DMSO-*d*_6_, 25°C) *δ* = 169.26, 156.62, 149.93, 143.65, 141.11, 139.65, 133.74, 130.22, 130.15, 128.27, 126.64, 125.95, 124.07, 123.84, 123.56, 122.77, 121.23, 119.59, 119.40, 119.00, 118.98, 118.93, 118.66, 117.94, 111.77, 40.39, 40.26, 40.12, 39.98, 39.84, 39.70, 39.56, 14.82, 0.59 ppm; MS (ESI) *m/z* 548.38 = [M + H]^+^, HRMS *m/z* 548.1633 = [M + H]^+^, calcd. for C_32_H_25_N_3_O_4_S *m/z* = 547.1566.

*N*-(4-(*N*-(6-Chloropyridin-3-yl)sulfamoyl)phenyl)-1-methyl-9*H*-carbazole-2-carboxamide (**7b**): brown solid; yield: 56%; m. p. 93°C; ^1^H NMR (600 MHz, DMSO-*d*_6_, 25°C) *δ* = 11.41 (s, 1H), 10.76 (s, 1H), 10.68 (s, 1H), 8.16 (d, *J* = 7.7 Hz, 1H), 8.13 (d, *J* = 2.9 Hz, 1H), 8.06 (d, *J* = 8.0 Hz, 1H), 7.99—7.94 (m, 2H), 7.78 (d, *J* = 8.9 Hz, 2H), 7.59 (dd, *J* = 8.7, 2.9 Hz, 1H), 7.55 (d, *J* = 8.1 Hz, 1H), 7.44 (d, *J* = 8.5 Hz, 2H), 7.31 (d, *J* = 8.0 Hz, 1H), 7.22—7.17 (m, 1H), 2.62 (s, 3H) ppm; MS (ESI) *m/z* 491.45 = [M + H]^+^, HRMS *m/z* 491.0922 = [M + H]^+^, calcd. for C_25_H_19_ClN_4_O_3_S *m/z* = 490.0866.

*N*'-Benzoyl-*N*'-(tert-butyl)-1-methyl-9*H*-carbazole-2-carbohydrazide (**7c**): white solid; yield: 79%; m. p. 212°C; ^1^H NMR (600 MHz, DMSO-*d*_6_, 25°C) *δ* = 11.23 (s, 1H), 10.53 (s, 1H), 8.08 (d, *J* = 7.8 Hz, 1H), 7.87 (d, *J* = 8.0 Hz, 1H), 7.51—7.47 (m, 3H), 7.44—7.36 (m, 4H), 7.16—7.13 (m, 1H), 6.52 (d, *J* = 8.0 Hz, 1H), 2.08 (s, 3H), 1.55 (s, 9H) ppm; ^13^C NMR (151 MHz, DMSO-*d*_6_, 25°C) *δ* = 172.62, 168.54, 140.91, 139.46, 138.59, 131.17, 129.71, 128.01, 127.37, 126.62, 123.45, 122.63, 121.14, 119.33, 119.21, 117.70, 117.56, 111.68, 60.59, 28.06, 13.74 ppm; MS (ESI) *m/z* 422.40 = [M + Na]^+^, HRMS *m/z* 400.2014 = [M + H]^+^, calcd. for C_25_H_25_N_3_O_2_ *m/z* = 399.1947.

*N*-(tert-Butyl)-4-methyl-*N*'-(1-methyl-9*H*-carbazole-2-carbonyl)benzenesulfonohydrazide (**7d**): yellow solid; yield: 85%; m. p. 97°C; ^1^H NMR (600 MHz, DMSO-*d*_6_, 25°C) *δ* = 11.33 (s, 1H), 10.31 (s, 1H), 8.14 (d, *J* = 7.7 Hz, 1H), 8.02—8.00 (m, 3H), 7.54 (d, *J* = 7.9 Hz, 1H), 7.44—7.40 (m, 3H), 7.20—7.13 (m, 2H), 2.39 (s, 3H), 1.40 (d, *J* = 3.2 Hz, 9H) ppm; ^13^C NMR (151 MHz, DMSO-*d*_6_, 25°C) *δ* = 169.98, 143.70, 141.00, 139.59, 139.32, 131.78, 129.74, 128.36, 126.58, 123.51, 122.76, 121.15, 119.34, 118.74, 117.59, 111.73, 63.29, 28.79, 21.50, 14.51 ppm; MS (ESI) *m/z* 450.23 = [M + H]^+^, HRMS *m/z* 450.1836 = [M + H]^+^, calcd. for C_25_H_27_N_3_O_3_S *m/z* = 449.1773.

1-Methyl-*N*-(2-oxo-2-phenylethyl)-9*H*-carbazole-2-carboxamide (**7e**): yellow solid; yield: 67%; m. p. 153°C; ^1^H NMR (600 MHz, DMSO-*d*_6_) *δ* = 11.32 (s, 1H), 8.59 (t, *J* = 5.7 Hz, 1H), 8.15—8.00 (m, 4H), 7.72—7.67 (m, 1H), 7.61—7.53 (m, 3H), 7.44—7.41 (m, 1H), 7.28 (d, *J* = 8.0 Hz, 1H), 7.19—7.17 (m, 1H), 4.80 (d, *J* = 5.8 Hz, 2H), 2.66 (s, 3H) ppm; ^13^C NMR (151 MHz, DMSO) *δ* = 196.02, 170.49, 140.97, 139.75, 135.63, 134.01, 133.87, 129.32, 128.37, 126.38, 123.12, 122.88, 121.05, 119.28, 119.03, 118.63, 117.69, 111.70, 46.92, 14.75 ppm; MS (ESI) *m/z* 343.33 = [M + H]^+^, HRMS *m/z* 343.1469 = [M + H]^+^, calcd. for C_22_H_18_N_2_O_2_ *m/z* = 342.1368.

2-(6-Chloro-9*H*-carbazol-2-yl)-*N*-(2-(methylsulfonamido)phenyl)propanamide (**11a**): yellow solid; yield: 64%; m. p. 163°C; ^1^H NMR (600 MHz, DMSO-*d*_6_, 25°C) *δ* = 11.39 (s, 1H), 9.56 (s, 1H), 8.79 (s, 1H), 8.18 (d, *J* = 2.1 Hz, 1H), 8.11 (d, *J* = 8.1 Hz, 1H), 7.64 (dd, *J* = 8.1, 1.5 Hz, 1H), 7.54—7.51 (m, 1H), 7.48 (dd, *J* = 8.5, 0.5 Hz, 1H), 7.36 (dd, *J* = 8.6, 2.1 Hz, 1H), 7.33 (dd, *J* = 7.9, 1.6 Hz, 1H), 7.25—7.22 (m, 2H), 7.17 (td, *J* = 7.7, 1.6 Hz, 1H), 4.07 (q, *J* = 7.0 Hz, 1H), 2.64 (s, 3H), 1.53 (d, *J* = 7.0 Hz, 3H) ppm; MS (ESI) *m/z* 442.48 = [M + H]^+^, HRMS *m/z* 442.0985 = [M + H]^+^, calcd. for C_22_H_20_ClN_3_O_3_S *m/z* = 441.0914.

2-(6-Chloro-9*H*-carbazol-2-yl)-*N*-(2-((dimethylphosphoryl)amino)phenyl)propanamide (**11b**): yellow solid; yield: 77%; m. p. 148°C; ^1^H NMR (600 MHz, DMSO-*d*_6_, 25°C) *δ* = 11.79 (s, 1H), 11.41 (s, 1H), 8.42 (dd, *J* = 8.5, 3.9 Hz, 1H), 8.17 (d, *J* = 2.1 Hz, 1H), 8.10 (d, *J* = 8.1 Hz, 1H), 7.53 (ddd, *J* = 13.8, 7.7, 1.6 Hz, 1H), 7.51—7.46 (m, 3H), 7.36 (dd, *J* = 8.6, 2.1 Hz, 1H), 7.20 (dd, *J* = 8.1, 1.5 Hz, 1H), 7.14—7.10 (m, 1H), 3.82 (q, *J* = 7.0 Hz, 1H), 1.77 (d, *J* = 13.6 Hz, 3H), 1.62 (d, *J* = 13.5 Hz, 3H), 1.54 (d, *J* = 7.0 Hz, 3H) ppm; ^13^C NMR (151 MHz, DMSO-*d*_6_, 25°C) *δ* = 172.56, 143.62, 143.60, 141.05, 140.28, 138.81, 132.93, 131.27, 131.20, 125.59, 124.04, 123.32, 121.21, 121.02, 120.15, 119.11, 112.82, 110.19, 48.74, 18.78 ppm; MS (ESI) *m/z* 425.38 = [M + H]^+^, HRMS *m/z* 425.1176 = [M + H]^+^, calcd. for C_23_H_23_ClN_3_O_2_P *m/z* = 424.1216.

2-(6-Chloro-9*H*-carbazol-2-yl)-*N*'-(p-tolyl)propanehydrazide (**11c**): white solid; yield: 88%; m. p. 188°C; ^1^H NMR (600 MHz, DMSO-*d*_6_, 25°C) *δ* = 11.38 (s, 1H), 9.83 (d, *J* = 3.2 Hz, 1H), 8.18 (d, *J* = 2.1 Hz, 1H), 8.09 (d, *J* = 8.1 Hz, 1H), 7.55 (d, *J* = 3.2 Hz, 1H), 7.51—7.46 (m, 2H), 7.36 (dd, *J* = 8.6, 2.2 Hz, 1H), 7.19 (dd, *J* = 8.2, 1.5 Hz, 1H), 6.84 (d, *J* = 8.1 Hz, 2H), 6.51—6.47 (m, 2H), 3.85 (q, *J* = 7.0 Hz, 1H), 2.12 (s, 3H), 1.46 (d, *J* = 7.0 Hz, 3H) ppm; ^13^C NMR (151 MHz, DMSO-*d*_6_, 25°C) *δ* = 173.63, 147.59, 141.02, 140.74, 138.80, 129.48, 127.40, 125.47, 124.14, 123.28, 120.87, 120.81, 120.12, 119.30, 112.80, 112.65, 110.13, 44.17, 20.54, 19.10 ppm; MS (ESI) *m/z* 378.18 = [M + H]^+^, HRMS *m/z* 378.1366 = [M + H]^+^, calcd. for C_22_H_20_ClN_3_O *m/z* = 377.1295.

2-(6-Chloro-9*H*-carbazol-2-yl)-*N*-(2-(methylsulfonyl)ethyl)propanamide (**11d**): white solid; yield: 73%; m. p. 149°C; ^1^H NMR (600 MHz, DMSO-*d*_6_, 25°C) *δ* = 11.38 (s, 1H), 8.31 (t, *J* = 5.7 Hz, 1H), 8.17 (d, *J* = 2.1 Hz, 1H), 8.06 (d, *J* = 8.1 Hz, 1H), 7.48 (d, *J* = 8.5 Hz, 1H), 7.43 (d, *J* = 1.4 Hz, 1H), 7.36 (dd, *J* = 8.6, 2.2 Hz, 1H), 7.13 (dd, *J* = 8.2, 1.5 Hz, 1H), 3.75 (q, *J* = 7.0 Hz, 1H), 3.47—3.40 (m, 2H), 3.25—3.17 (m, 2H) 2.90 (s, 3H), 1.41 (d, *J* = 7.0 Hz, 3H) ppm; ^13^C NMR (151 MHz, DMSO) *δ* = 174.26, 141.01, 140.95, 138.77, 125.46, 124.11, 123.26, 120.92, 120.75, 120.07, 119.26, 112.78, 110.12, 53.45, 46.02, 41.22, 33.35, 19.39 ppm; MS (ESI) *m/z* 378.90 = [M + H]^+^, HRMS *m/z* 379.0872 = [M + H]^+^, calcd. for C_18_H_19_ClN_2_O_3_S *m/z* = 378.0805.

2-(6-Chloro-9*H*-carbazol-2-yl)-*N*-(4-(*N*-(pyrimidin-2-yl)sulfamoyl)phenyl)propanamide (**11e**): yellow solid; yield: 65%; m. p. 157°C; ^1^H NMR (600 MHz, DMSO-*d*_6_, 25°C) *δ* = 11.37 (s, 1H), 10.49 (s, 1H), 8.46 (d, *J* = 4.9 Hz, 2H), 8.15 (d, *J* = 2.1 Hz, 1H), 8.08 (d, *J* = 8.1 Hz, 1H), 7.91—7.88 (m, 2H), 7.79—7.75 (m, 2H), 7.50—7.45 (m, 2H), 7.35 (dd, *J* = 8.6, 2.1 Hz, 1H), 7.20 (dd, *J* = 8.2, 1.5 Hz, 1H), 7.01 (t, *J* = 4.9 Hz, 1H), 4.00 (q, *J* = 6.9 Hz, 1H), 1.49 (d, *J* = 6.9 Hz, 3H) ppm; ^13^C NMR (151 MHz, DMSO) *δ* = 173.53, 157.37, 143.61, 141.02, 140.41, 138.80, 129.86, 129.33, 125.57, 124.05, 123.32, 121.11, 120.94, 120.12, 119.15, 118.93, 117.89, 112.81, 110.08, 46.99, 19.44 ppm; MS (ESI) *m/z* 506.31 = [M + H]^+^, HRMS *m/z* 506.1039 = [M + H]^+^, calcd. for C_25_H_20_ClN_5_O_3_S *m/z* = 505.0975.

### 4.6 General synthetic procedure for the target compounds **14a-g**

The general procedure for the synthesis of carbazole hydrazones **14a-g**: Various aldehyde (0.55 mmol) were added to solutions of substituted carbazole-based carbohydrazides **13** (0.5 mmol) in EtOH (6 mL), and then the mixtures were heated at reflux. After completion of reaction, the corresponding precipitation were collected by filtration, and washed with small amount of alcohol to give crude compounds, which were purified by flash chromatography and characterized by ESI-MS, HRMS, ^1^H NMR and ^13^C NMR spectra. Most of the obtained acylhydrazone derivatives are a mixed (E/Z) configuration, and so the NMR data of the mixture are linked with “and” in the follows.

2-(6-Chloro-9*H*-carbazol-2-yl)-*N*'-(2-hydroxy-3-methoxybenzylidene)propanehydrazide (**14a**): white solid; yield: 79%; m. p. 217°C; ^1^H NMR (600 MHz, DMSO-*d*_6_, 25°C) *δ* = 8.30 (s, 1H), 8.00—7.97 (m, 2H), 7.81—7.79 (m, 2H), 7.52—7.50 (m, 1H), 7.38 (d, *J* = 8.6 Hz, 1H), 7.31 (d, *J* = 2.0 Hz, 1H), 7.21 (dd, *J* = 8.2, 1.5 Hz, 1H), 7.02 (dd, *J* = 7.8, 1.4 Hz, 1H), 6.97 (dd, *J* = 8.1, 1.4 Hz, 1H), 6.84 (t, *J* = 8.0 Hz, 1H), 3.89 (s, 1H), 3.86 (s, 3H), 1.63—1.61 (m, 3H) ppm; ^13^C NMR (151 MHz, DMSO-*d*_6_, 25°C) *δ* = 170.11, 148.34, 147.44 and 147.40, 146.30, 141.04, 140.35, 138.81, 125.57, 124.07, 123.32, 121.10, 120.95, 120.14, 119.43, 119.32, 119.25, 114.15, 113.23 and 112.75, 112.82, 110.11, 56.25, 44.83, 19.36 ppm; MS (ESI) *m/z* 422.41 = [M + H]^+^, HRMS *m/z* 422.1257 = [M + H]^+^, calcd. for C_23_H_20_ClN_3_O_3_ *m/z* = 421.1193.

2-(6-Chloro-9*H*-carbazol-2-yl)-*N*'-(3-(4-chlorophenyl)allylidene)propanehydrazide (**14b**): white solid; yield: 82%; m. p. 186°C; ^1^H NMR (600 MHz, DMSO-*d*_6_, 25°C) *δ* = 11.46 and 11.32 (s, 1H), 11.37 and 11.24 (s, 1H), 8.17 and 8.15 (d, *J* = 2.1 Hz, 1H), 8.09 and 8.07 (d, 8.1 Hz, 1H), 7.96 and 7.77 (dd, *J* = 6.0, 2.4 Hz, 1H), 7.62 (dd, *J* = 10.1, 7.8 Hz, 2H), 7.49—7.41 (m, 4H), 7.35 (td, *J* = 8.8, 2.2 Hz, 1H), 7.17 (dt, *J* = 8.2, 1.4 Hz, 1H), 7.00—6.95 (m, 2H), 1.46 (t, *J* = 7.3 Hz, 3H) ppm; ^13^C NMR (151 MHz, DMSO-*d*_6_, 25°C) *δ* = 170.26, 148.69, 141.02, 140.58, 138.80, 137.26, 130.13, 129.29, 129.18, 129.14, 126.87, 121.04, 120.93, 120.12, 112.81, 110.07, 44.89, 40.40, 40.27, 40.13, 39.99, 39.85, 39.71, 39.57, 29.55, 29.49, 29.04, 22.56, 19.45 and 19.07 ppm; MS (ESI) *m/z* 436.34 = [M + H]^+^, HRMS *m/z* 436.0976 = [M + H]^+^, calcd. for C_24_H_19_Cl_2_N_3_O *m/z* = 435.0905.

*N*'-(Benzo[*d*][1,3]dioxol-5-ylmethylene)-2-(6-chloro-9*H*-carbazol-2-yl)propanehydrazide (**14c**): white solid; yield: 84%; m. p. 240°C; ^1^H NMR (600 MHz, DMSO-*d*_6_, 25°C) *δ* = 11.45 and 11.37 (s, 1H), 11.34 and 11.18 (s, 1H), 8.15 and 814 (d, *J* = 2.1 Hz, 1H), 8.11 and 7.82 (s, 1H), 8.07 and 8.06 (d, *J* = 8.1 Hz, 1H), 7.49—7.44 (m, 2H), 7.35 (m, 1H), 7.29 and 7.21 (d, *J* = 1.6 Hz, 1H), 7.20—7.18 (m, 1H), 7.11 and 7.06 (dd, *J* = 8.0, 1.6 Hz, 1H), 6.95 (d, *J* = 5.5 Hz, 1H), 6.09 (d, *J* = 1.0 Hz, 1H), 6.07 (d, *J* = 1.0 Hz, 1H), 4.82 and 3.83 (q, *J* = 7.0 Hz, 1H), 1.47 (d, *J* = 7.0 Hz, 3H) ppm; ^13^C NMR (151 MHz, DMSO-*d*_6_, 25°C) *δ* = 175.43 and 170.17, 149.44 and 149.19, 148.44 and 148.38, 146.83 and 142.78, 141.20 and 141.06, 141.03 and 140.63, 138.80 and 138.73, 129.25 and 129.14, 125.53 and 125.43, 124.10 and 124.09, 123.57 and 123.34, 123.30 and 123.24, 121.01 and 120.96, 120.87 and 120.64, 120.11 and 120.04, 119.61 and 119.26, 112.80 and 112.74, 110.15 and 110.08, 108.88, 105.53 and 105.29, 101.97 and 101.93, 44.90 and 41.53, 19.49 and 19.38 ppm; MS (ESI) *m/z* 442.01 = [M + Na]^+^, HRMS *m/z* 420.1110 = [M + H]^+^, calcd. for C_23_H_18_ClN_3_O_3_ *m/z* = 419.1037.

*N*'-((1*H*-Indol-3-yl)methylene)-2-(6-chloro-9*H*-carbazol-2-yl)propanehydrazide (**14d**): white solid; yield: 78%; m. p. 209°C; ^1^H NMR (600 MHz, DMSO-*d*_6_, 25°C) *δ* = 11.52 and 11.49 (d, *J* = 2.7 Hz, 1H), 11.37 and 11.32 (s, 1H), 11.20 and 11.00 (s, 1H), 8.36 and 8.13 (s, 1H), 8.21—8.11 (m, 2H), 8.09—8.05 (d, *J* = 8.1 Hz, 1H), 7.75 and 7.71 (d, *J* = 2.8 Hz, 1H), 7.52 and 7.50 (s, 1H), 7.47 and 7.44 (d, *J* = 0.5 Hz, 1H), 7.43—7.40 (m, 1H), 7.35 and 7.32 (dd, *J* = 8.6, 2.2 Hz, 1H), 7.27—7.20 (m, 2H), 7.19—7.09 (m, 1H), 4.91 and 3.84 (q, *J* = 7.0 Hz, 1H), 1.51 (d, *J* = 3.5 Hz, 3H) ppm; ^13^C NMR (151 MHz, DMSO-*d*_6_, 25°C) *δ* = 174.75 and 169.51, 144.17 and 141.37, 141.07 and 141.05, 141.02 and 140.76, 138.80 and 138.73, 137.47 and 137.44, 130.59 and 130.54, 125.48 and 125.39, 124.74 and 124.51, 124.12, 123.27 and 123.21, 123.01, 122.35 and 122.09, 120.95 and 120.80, 120.73 and 120.61, 120.09 and 120.00, 119.57 and 119.33, 112.78 and 112.71, 112.30 and 112.23, 112.05 and 111.96, 110.10 and 110.06, 44.88 and 41.46, 19.75 and 19.49 ppm; MS (ESI) *m/z* 415.26 = [M + H]^+^, HRMS *m/z* 415.1324 = [M + H]^+^, calcd. for C_24_H_19_ClN_4_O *m/z* = 414.1247.

2-(6-Chloro-9*H*-carbazol-2-yl)-*N*'-(4-hydroxy-3-methoxybenzylidene)propanehydrazide (**14e**): white solid; yield: 90%; m. p. 239°C; ^1^H NMR (600 MHz, DMSO-*d*_6_, 25°C) *δ* = 11.37 and 11.30 (s, 1H), 11.36 and 11.12 (s, 1H), 9.49 and 9.45 (s, 1H), 8.17—6.78 (m, 10H), 4.78 and 3.82 (q, *J* = 7.0 Hz, 1H), 3.86 and 3.79 (s, 3H), 1.48 and 1.45 (d, *J* = 7.0 Hz, 3H) ppm; ^13^C NMR (151 MHz, DMSO-*d*_6_, 25°C) *δ* = 175.23 and 170.00, 149.32 and 148.95, 148.43, 147.54 and 143.19, 141.47 and 140.74, 141.07 and 141.03, 138.80 and 138.73, 126.27 and 126.12, 125.52 and 125.43, 124.13 and 124.09, 123.30 and 123.25, 122.41 and 121.65, 120.99, 120.84 and 120.61, 120.11 and 120.03, 119.71 and 119.25, 115.87 and 115.83, 112.80 and 112.72, 110.05 and 110.03, 109.35 and 109.27, 56.00 and 55.97, 44.87 and 41.85, 19.58 and 19.42 ppm; MS (ESI) *m/z* 422.13 = [M + H]^+^, HRMS *m/z* 422.1266 = [M + H]^+^, calcd. for C_23_H_20_ClN_3_O_3_ *m/z* = 421.1193.

2-(6-Chloro-9*H*-carbazol-2-yl)-*N*'-(4-chlorobenzylidene)propanehydrazide (**14f**): white solid; yield: 85%; m. p. 218°C; ^1^H NMR (600 MHz, DMSO-*d*_6_, 25°C) *δ* = 11.61 and 11.35 (s, 1H), 11.37 and 11.32 (s, 1H), 8.19 and 7.89 (s, 1H), 8.16 and 8.13 (d, *J* = 2.1 Hz, 1H), 8.09 and 8.05 (d, *J* = 8.1 Hz, 1H), 7.71 and 7.68 (s, 1H), 7.70 and 7.67 (s, 1H), 7.51—7.44 (m, 4H), 7.37—7.33 (m, 1H), 7.20—7.18 (m, 1H), 4.82 and 3.86 (q, *J* = 7.0 Hz, 1H), 1.48 (d, *J* = 7.0 Hz, 3H) ppm; ^13^C NMR (151 MHz, DMSO-*d*_6_, 25°C) *δ* = 175.64 and 170.44, 145.71, 141.70, 141.03, 140.94 and 140.48, 138.81 and 138.73, 134.82 and 134.52, 133.77 and 133.71, 129.37 and 129.33, 129.06 and 128.81, 125.55 and 125.47, 124.08, 123.31 and 123.26, 121.05 and 121.01, 120.91 and 120.68, 120.13 and 120.05, 119.54 and 119.25, 112.81 and 112.76, 110.32 and 110.10, 44.93, 41.63, 19.38 and 19.35 ppm; MS (ESI) *m/z* 410.15 = [M + H]^+^, HRMS *m/z* 410.0798 = [M + H]^+^, calcd. for C_22_H_17_ClN_3_O *m/z* = 409.0749.

2-(6-Chloro-9*H*-carbazol-2-yl)-*N*'-(2,4-dichlorobenzylidene)propanehydrazide (**14g**): white solid; yield: 82%; m. p. 241°C; ^1^H NMR (600 MHz, DMSO-*d*_6_, 25°C) *δ* = 11.83 and 11.53 (s, 1H), 11.38 and 11.32 (s, 1H), 8.54 and 8.24 (s, 1H), 8.17 and 8.13 (d, *J* = 2.1 Hz, 1H), 8.10 and 8.06 (d, *J* = 8.1 Hz, 1H), 8.02 and 7.91 (d, *J* = 8.5 Hz, 1H), 7.69 and 7.67 (d, *J* = 2.1 Hz, 1H), 7.54—7.45 (m, 3H), 7.37—7.33 (m, 1H), 7.19 and 7.18 (d, *J* = 1.5 Hz, 1H), 4.81 and 3.85 (q, *J* = 7.0 Hz, 1H), 1.48 (d, *J* = 7.0 Hz, 3H) ppm; ^13^C NMR (151 MHz, DMSO-*d*_6_, 25°C) *δ* = 175.76 and 170.56, 141.80 and 141.03, 140.83 and 140.29, 138.82 and 138.74, 137.98, 135.42 and 135.12, 134.16 and 133.96, 131.14 and 131.07, 129.81 and 129.79, 128.47, 128.43 and 128.30, 125.58 and 125.50, 124.06, 123.33 and 123.28, 121.12 and 121.07, 120.96 and 120.71, 120.14 and 120.06, 119.49 and 119.21, 112.83 and 112.78, 110.30 and 110.13, 45.11 and 41.73, 19.38 and 19.36 ppm; MS (ESI) *m/z* 443.92 = [M + H]^+^, HRMS *m/z* 444.0430 = [M + H]^+^, calcd. for C_22_H_16_Cl_3_N_3_O *m/z* = 443.0359.

### 4.7 Crystal structure determination of **14a**

Crystal Data for C_23_H_20_ClN_3_O_3_ (*M* = 421.87 g/mol): triclinic, space group P-1 (no. 2), *a* = 10.7893 (14) Å, *b* = 13.0199 (15) Å, *c* = 15.497 (2) Å, *α* = 111.749 (12)°, *β* = 100.232 (11)°, *γ* = 90.146 (10)°, *V* = 1984.2 (5) Å^3^, *Z* = 4, *T* = 149.99 (10) K, μ(Cu Kα) = 1.966 mm^−1^, *Dcalc* = 1.412 g/cm^3^, 13,393 reflections measured (7.33° ≤ 2Θ ≤ 148.834°), 7661 unique (*R*_int_ = 0.1060, *R*_sigma_ = 0.1452) which were used in all calculations. The final *R*_1_ was 0.1024 (I > 2σ(I)) and *wR*_2_ was 0.2727 (all data).

### 4.8 *In vitro* cytotoxicity assays

The *in vitro* cytotoxicity of these carbazole derivatives against human melanoma (A875), human gastric adenocarcinoma cells (7901), and a subclone of African green monkey kidney cell line MA-104 (MARC145) cell lines was evaluated using the MTT assay. Briefly, A875, 7901, and MARC145 cells were seeded at 2 × 104 cells per well in 96-well plates and grown to subconfluence. After removal of the growth medium, six serial dilutions of each tested compound in 200 µL test medium were added. Plates were incubated at 37°C in a humidified atmosphere containing 5% CO_2_. After 72 h of exposure, the culture medium was removed and 30 µL of the MTT solution (5 mg/mL in PBS) was added to each well. The plate was further incubated for 4 h to allow MTT formazan formation. To dissolve the resulting MTT formazan, 50 µL of DMSO was added to each well, followed by thorough mixing with a microplate shaker. Absorbance at 570 nm was measured on a microplate reader (Thermo Scientific, MK3). All data were analyzed with SPSS software, and the 50% inhibitory concentrations (IC_50_) of each compound for the different cell lines were determined. All assays were performed in triplicate on three independent experiments.

## Data Availability

The original contributions presented in the study are included in the article/Supplementary Material, further inquiries can be directed to the corresponding authors.
