# Supplementary material for: Structural diversity-guided optimization of carbazole derivatives as potential cytotoxic agents
Source: Front Chem. 2023 Jan 18;11:1104868. doi: 10.3389/fchem.2023.1104868 (PMC9890180; doi:10.3389/fchem.2023.1104868)

**Structural diversity-guided optimization of carbazole derivatives as potential cytotoxic agents**

Zilin Gao^a,b^, Yu Chen^a^, Yufei Nie^a^, Keming Chen^a^, Xiufang Cao^a,^*, Shaoyong Ke^b,^*

*^a^College of Science, Huazhong Agricultural University, Wuhan 430070, China*

*^b^National Biopesticide Engineering Research Centre, Hubei Biopesticide Engineering Research Centre, Hubei Academy of Agricultural Sciences, Wuhan 430064, China*

**Supporting Information**

**Experimental section**

***Instrumentation and chemicals***

^1^H NMR and ^13^C NMR spectra were recorded on a Bruker Avance III 600 MHz FT-NMR spectrometer using DMSO-*d*_6_ as the solvent and tetramethylsilane (TMS) as the internal standard. Mass spectra were performed on a WATERS ACQUITY UPLC^®^ H-CLASS PDA (Waters^®^) instrument. Thin-layer chromatography (TLC) was carried out on precoated silica gel plates GF254 (Qingdao Haiyang Chemical, China), and spots were visualized with ultraviolet light. All starting materials and reagents commercially available were used without further purification, unless otherwise specified.

***Crystal structure determination of 14a***

Crystal Data for C_23_H_20_ClN_3_O_3_ (*M*=421.87 g/mol): triclinic, space group P-1 (no. 2), *a* = 10.7893(14) Å, *b* = 13.0199(15) Å, *c* = 15.497(2) Å, *α* = 111.749(12)°, *β* = 100.232(11)°, *γ* = 90.146(10)°, *V*= 1984.2(5) Å^3^, *Z* = 4, *T* = 149.99(10) K, μ(Cu Kα) = 1.966 mm^-1^, *Dcalc* = 1.412 g/cm^3^, 13393 reflections measured (7.33° ≤ 2Θ ≤ 148.834°), 7661 unique (*R*_int_ = 0.1060, R_sigma_ = 0.1452) which were used in all calculations. The final *R*_1_ was 0.1024 (I > 2σ(I)) and *wR*_2_ was 0.2727 (all data).

**Table 1**Crystal data and structure refinement for compound 14a

| Identification code | 14a |
| --- | --- |
| Empirical formula | C_23_H_20_ClN_3_O_3_ |
| Formula weight | 421.87 |
| Temperature/K | 149.99(10) |
| Crystal system | triclinic |
| Space group | P-1 |
| a/Å | 10.7893(14) |
| b/Å | 13.0199(15) |
| c/Å | 15.497(2) |
| α/° | 111.749(12) |
| β/° | 100.232(11) |
| γ/° | 90.146(10) |
| Volume/Å^3^ | 1984.2(5) |
| Z | 4 |
| ρ_calc_g/cm^3^ | 1.412 |
| μ/mm^‑1^ | 1.966 |
| F(000) | 880.0 |
| Crystal size/mm^3^ | 0.14 × 0.12 × 0.1 |
| Radiation | Cu Kα (λ = 1.54184) |
| 2Θ range for data collection/° | 7.33 to 148.834 |
| Index ranges | -13 ≤ h ≤ 13, -10 ≤ k ≤ 16, -18 ≤ l ≤ 19 |
| Reflections collected | 13393 |
| Independent reflections | 7661 [R_int_ = 0.1060, R_sigma_ = 0.1452] |
| Data/restraints/parameters | 7661/7/547 |
| Goodness-of-fit on F^2^ | 0.883 |
| Final R indexes [I>=2σ (I)] | R_1_ = 0.1024, wR_2_ = 0.2450 |
| Final R indexes [all data] | R_1_ = 0.1749, wR_2_ = 0.2727 |
| Largest diff. peak/hole / e Å^-3^ | 1.53/-0.53 |

***Spectroscopy for target compounds***

Structures of target compounds were confirmed by their ^1^H NMR, ^13^C NMR, ESI-MS, and X-Ray analyses, and their ^1^H NMR, ^13^C NMR, ESI-MS were consistent with the assigned structures. The typical ^1^H NMR, ^13^C NMR for synthesized compounds have been presented in the following.


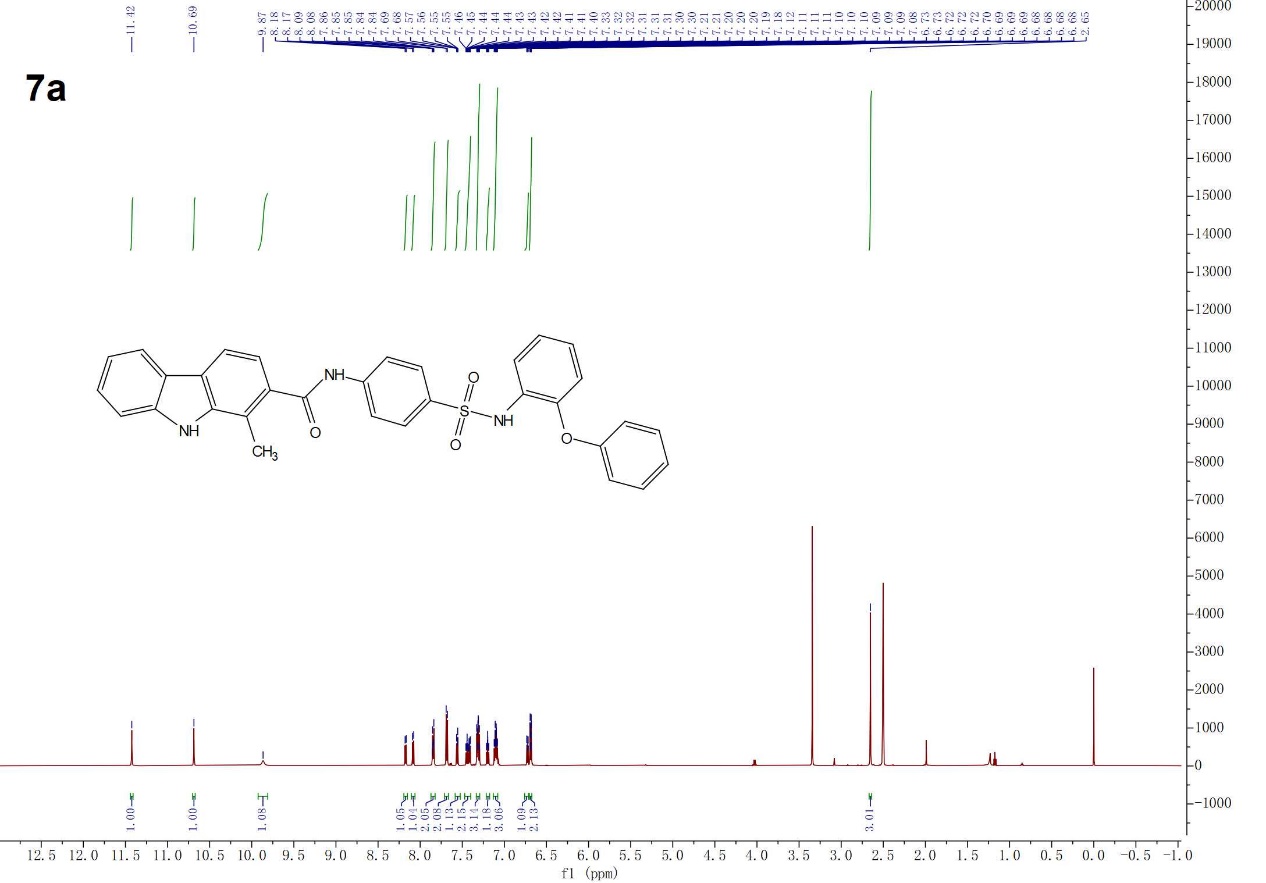

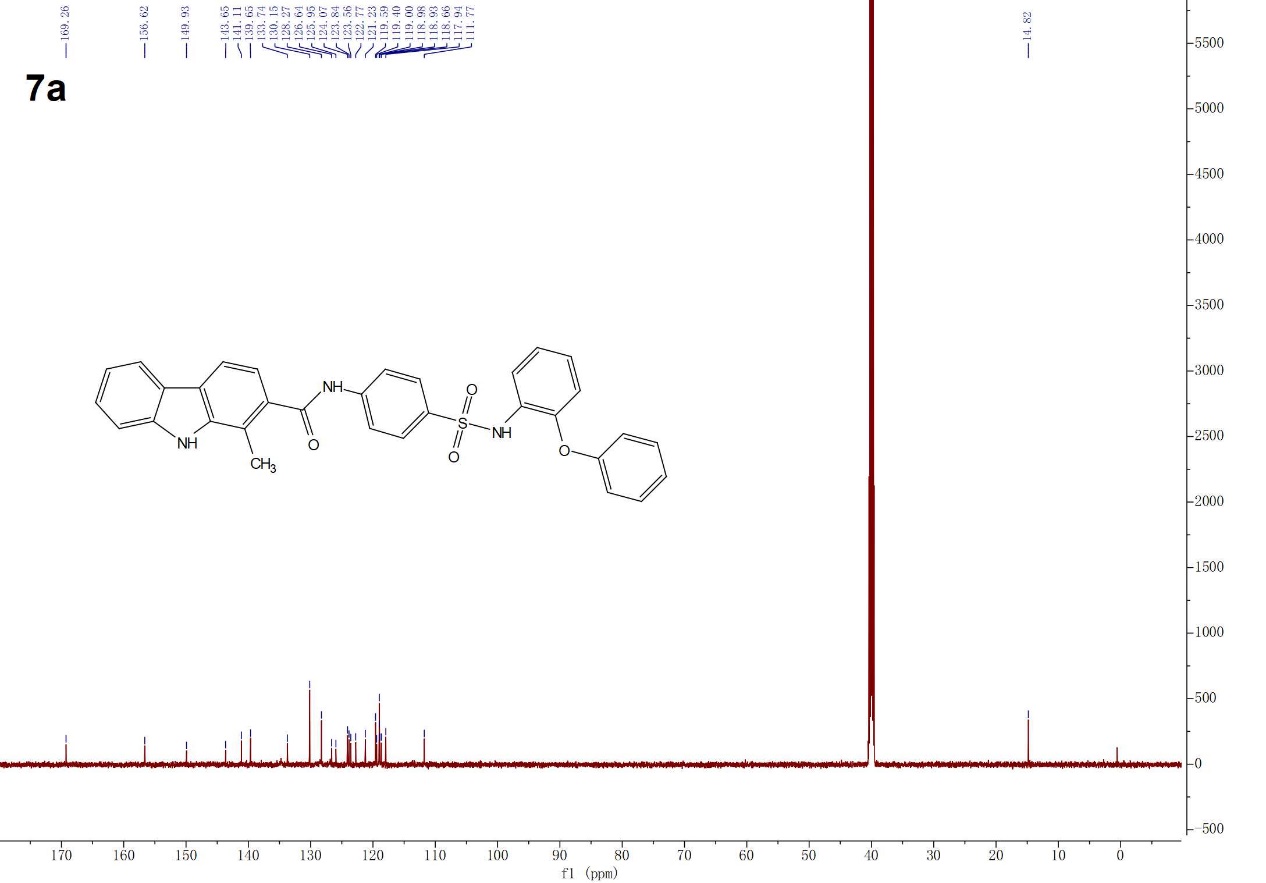


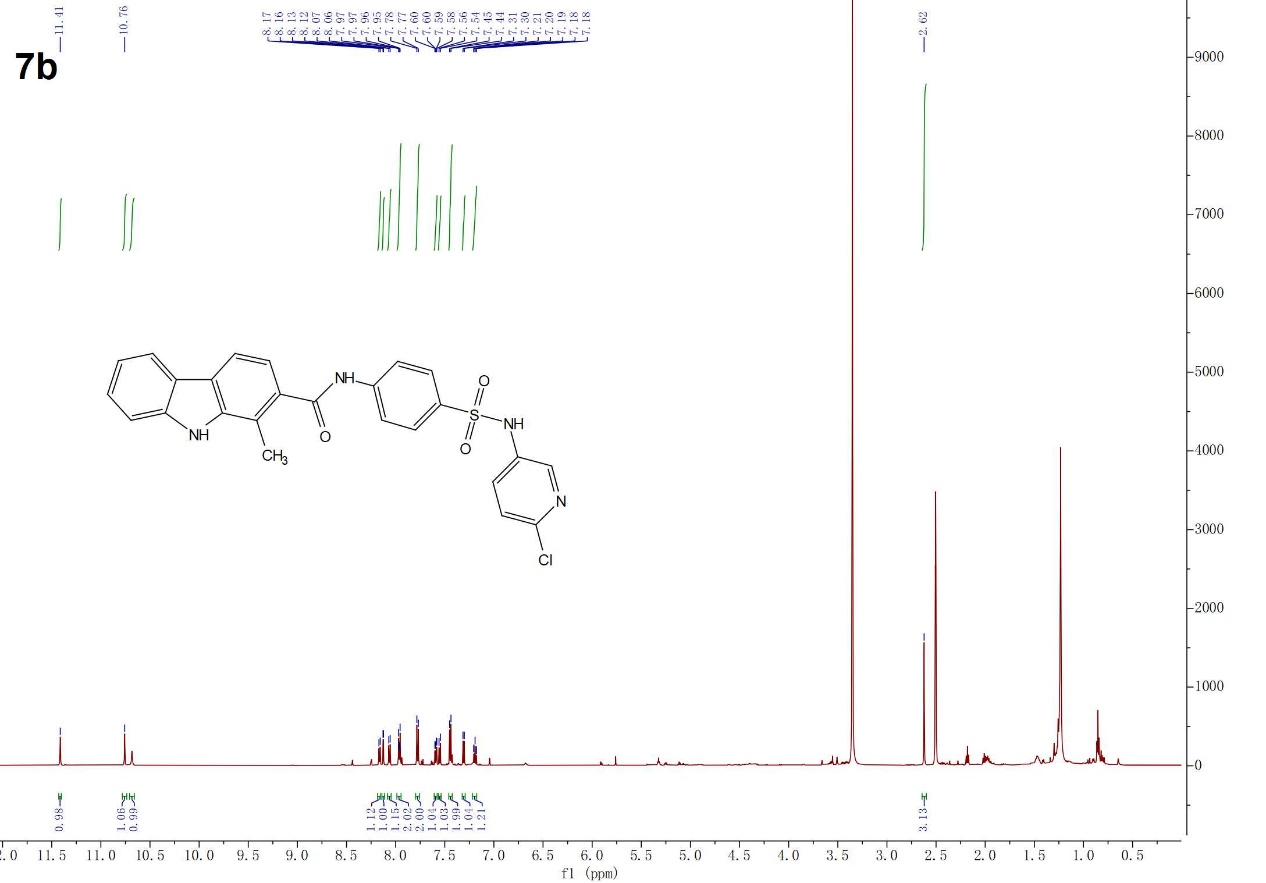


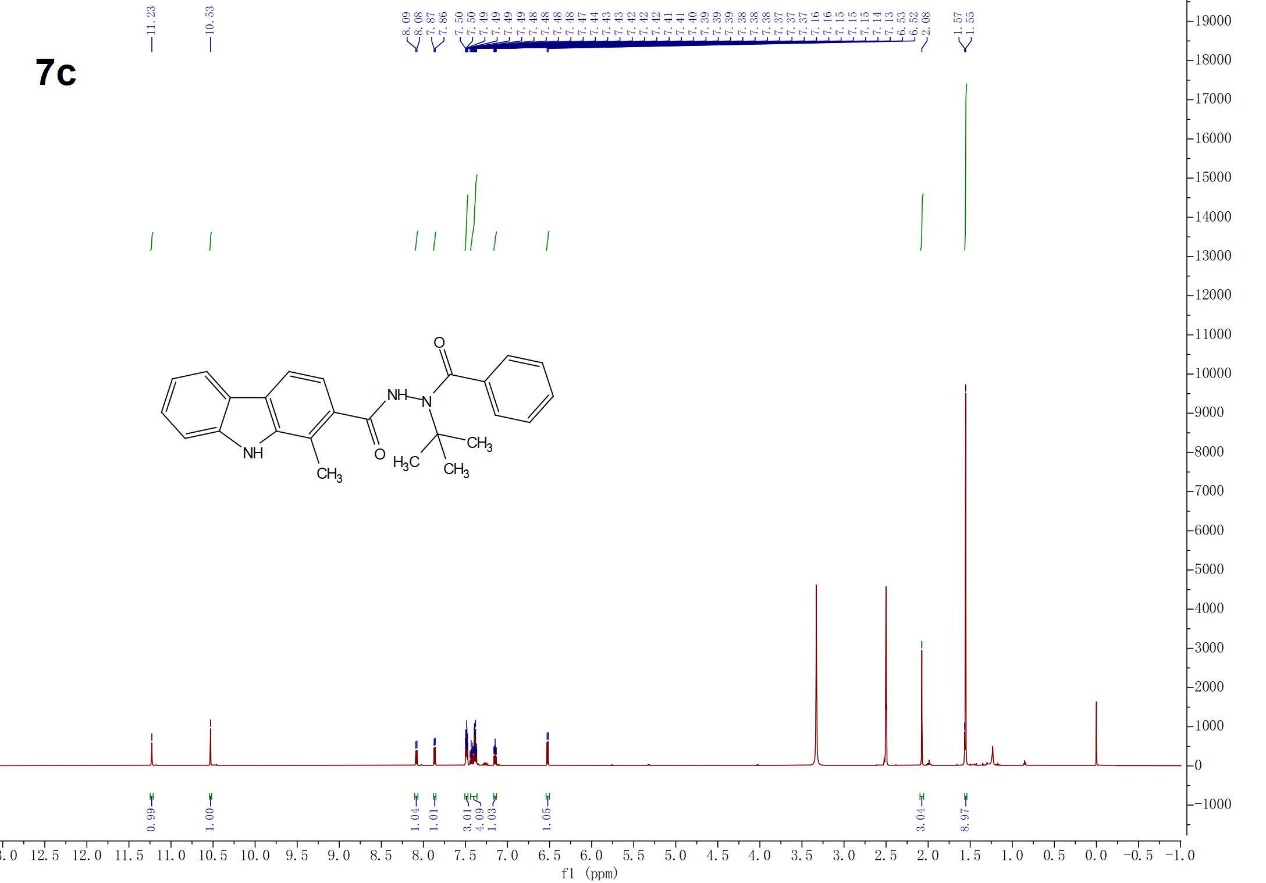

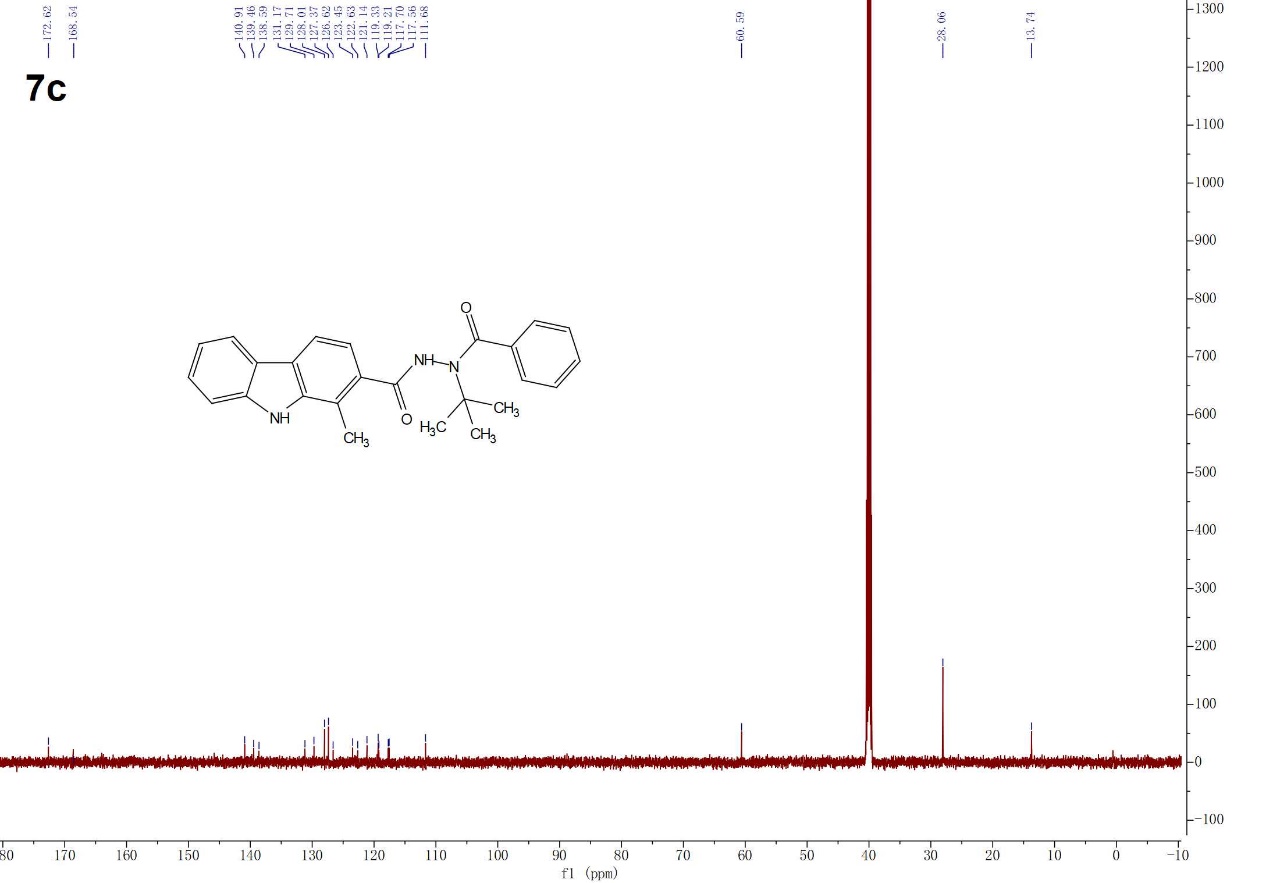


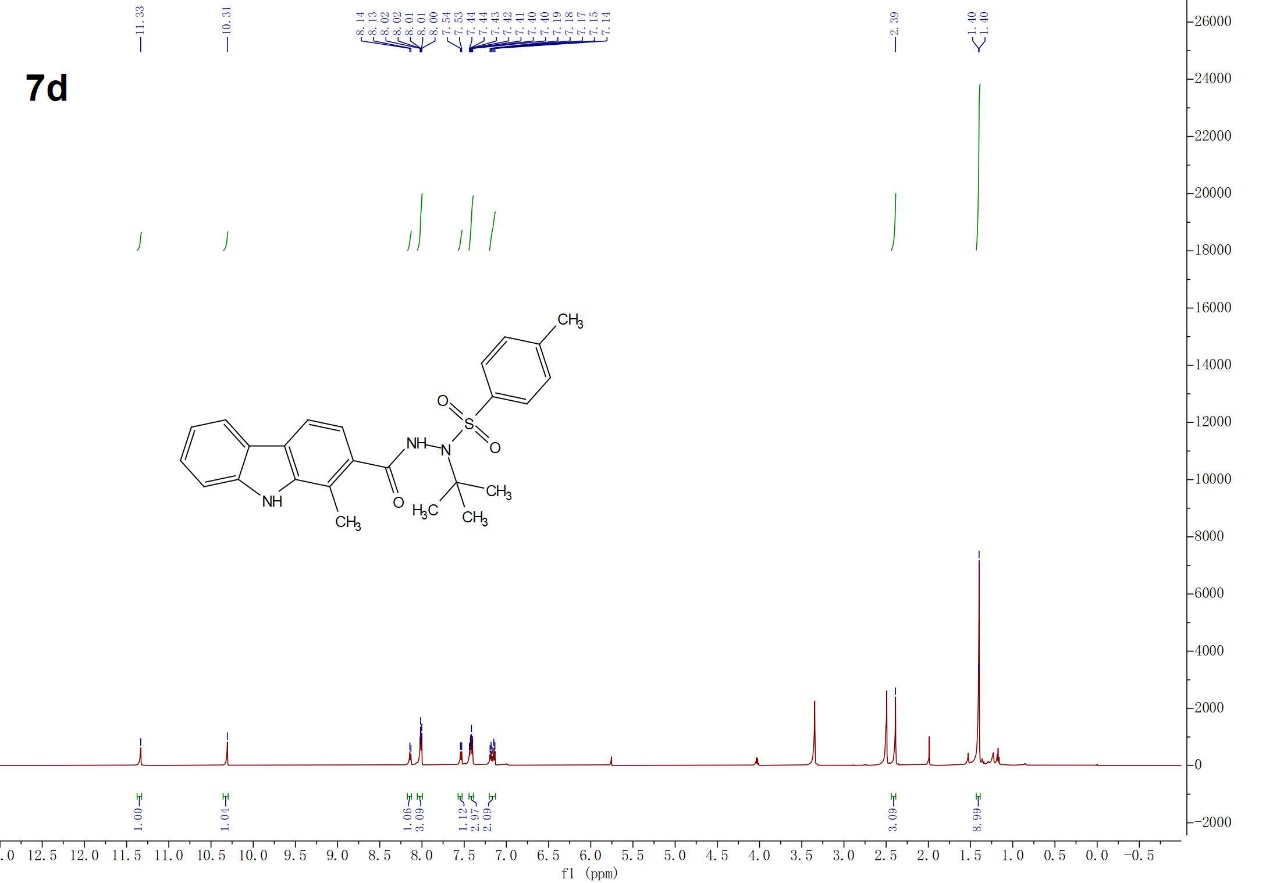

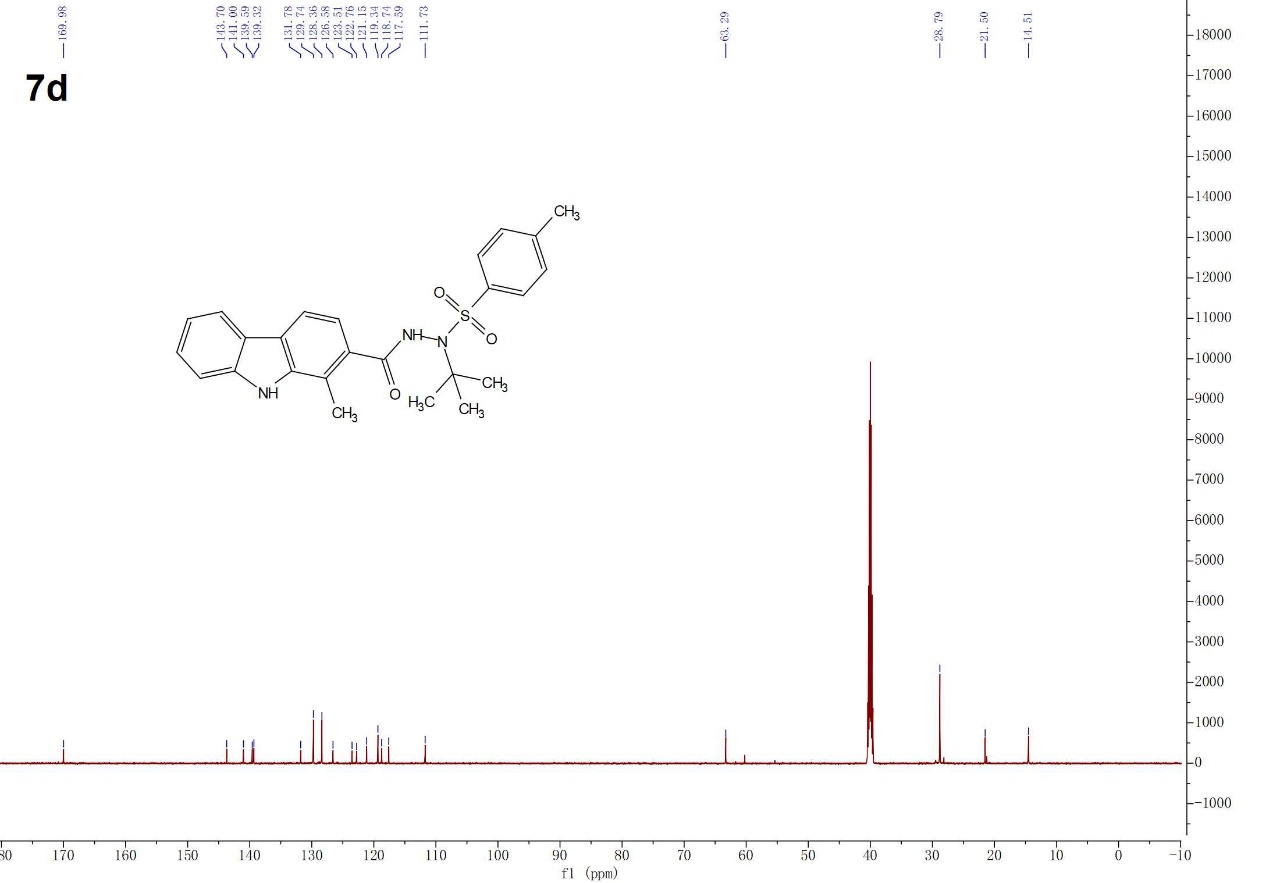


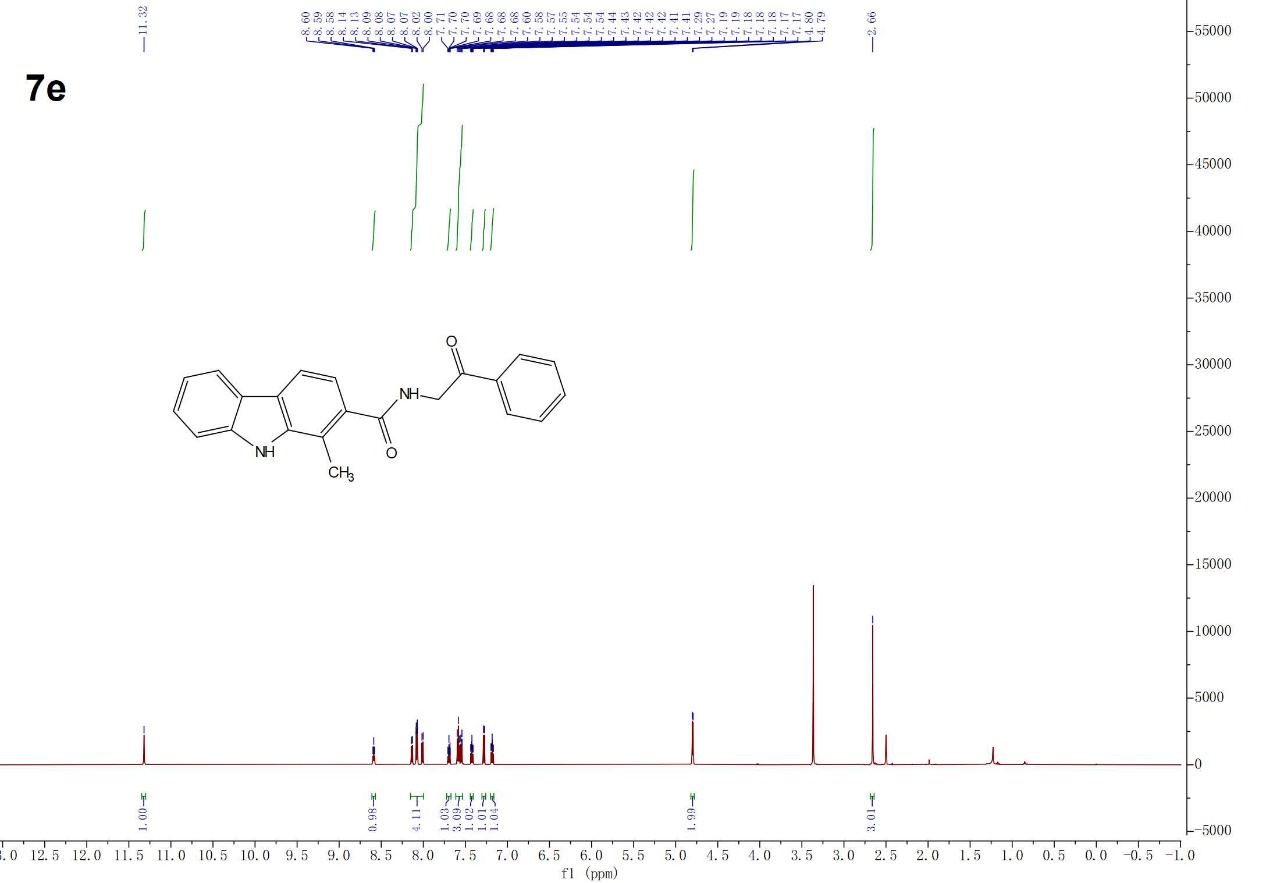

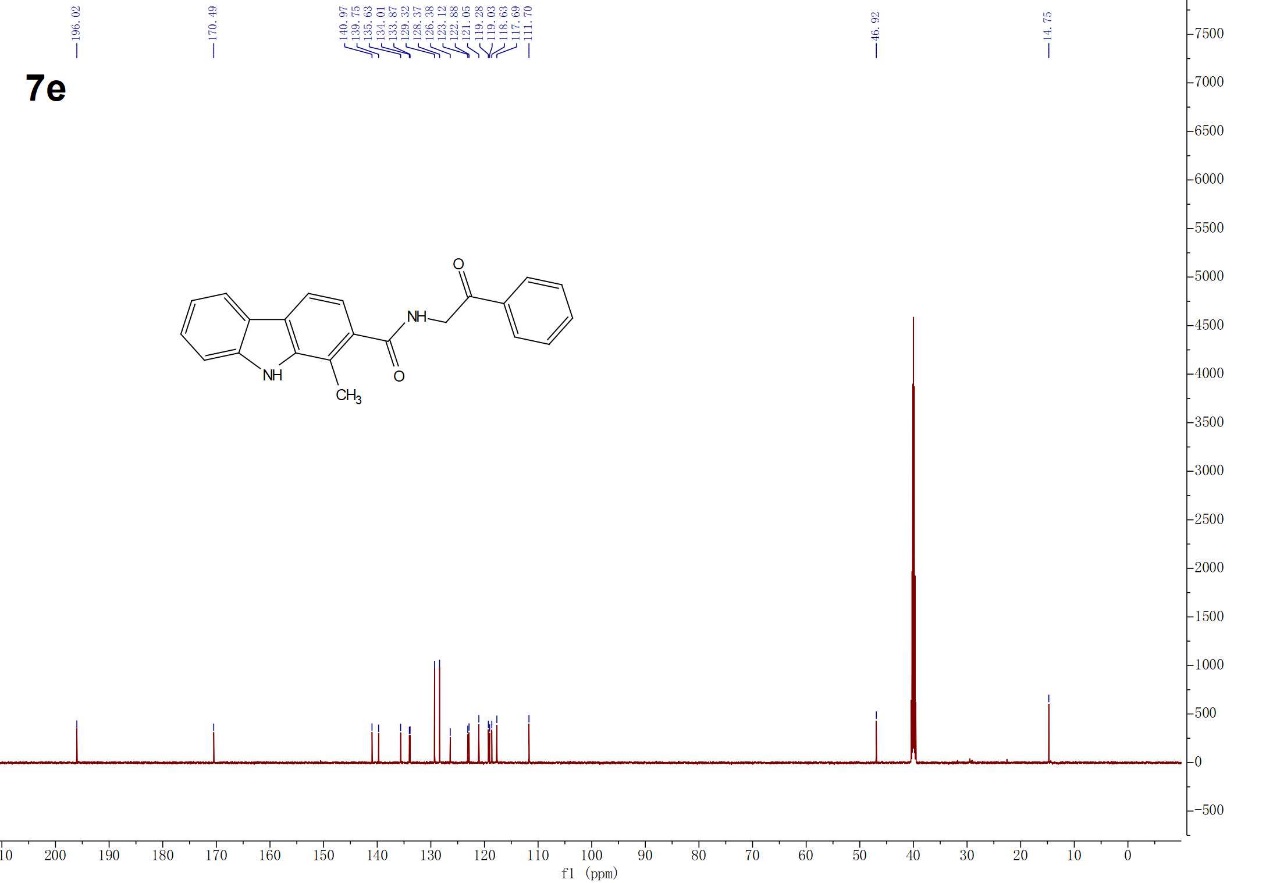


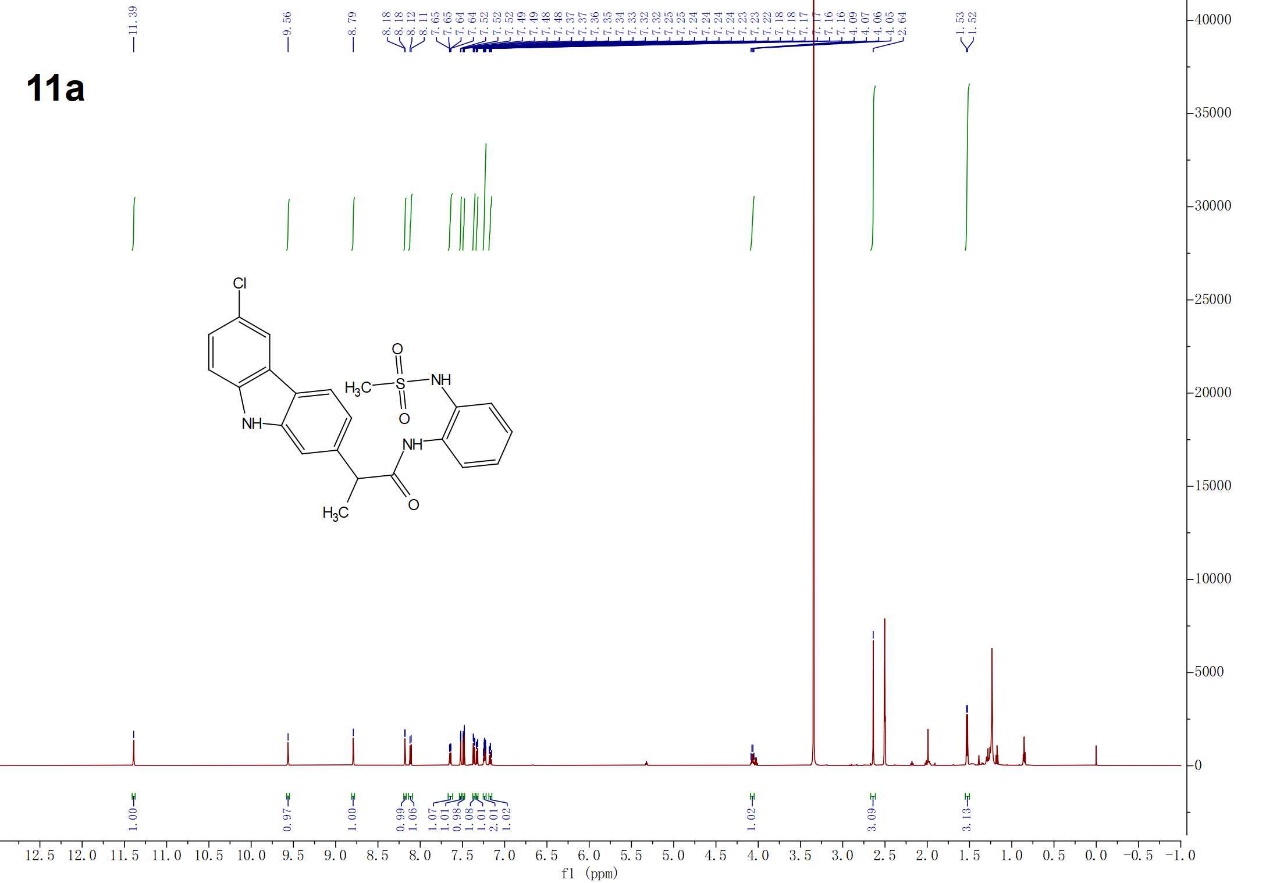

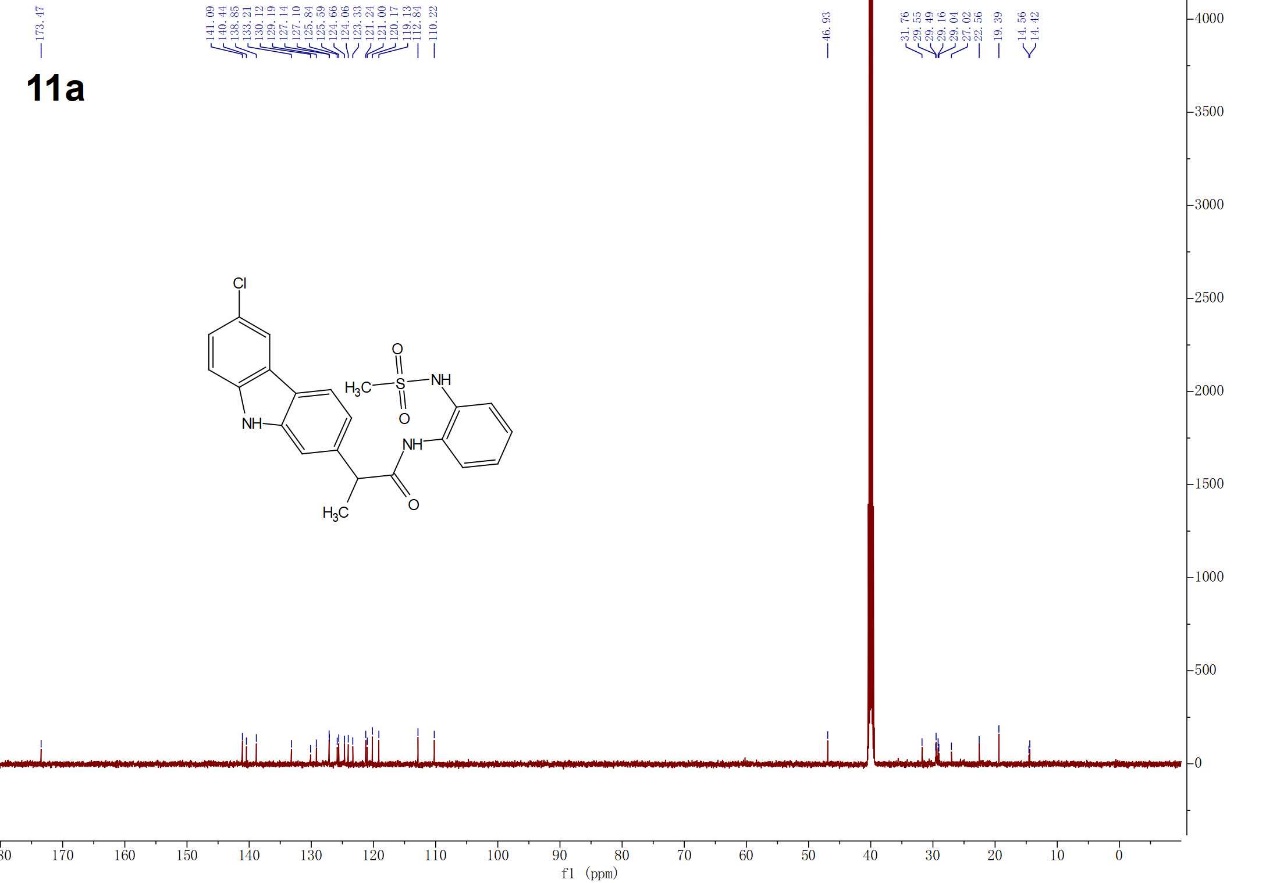


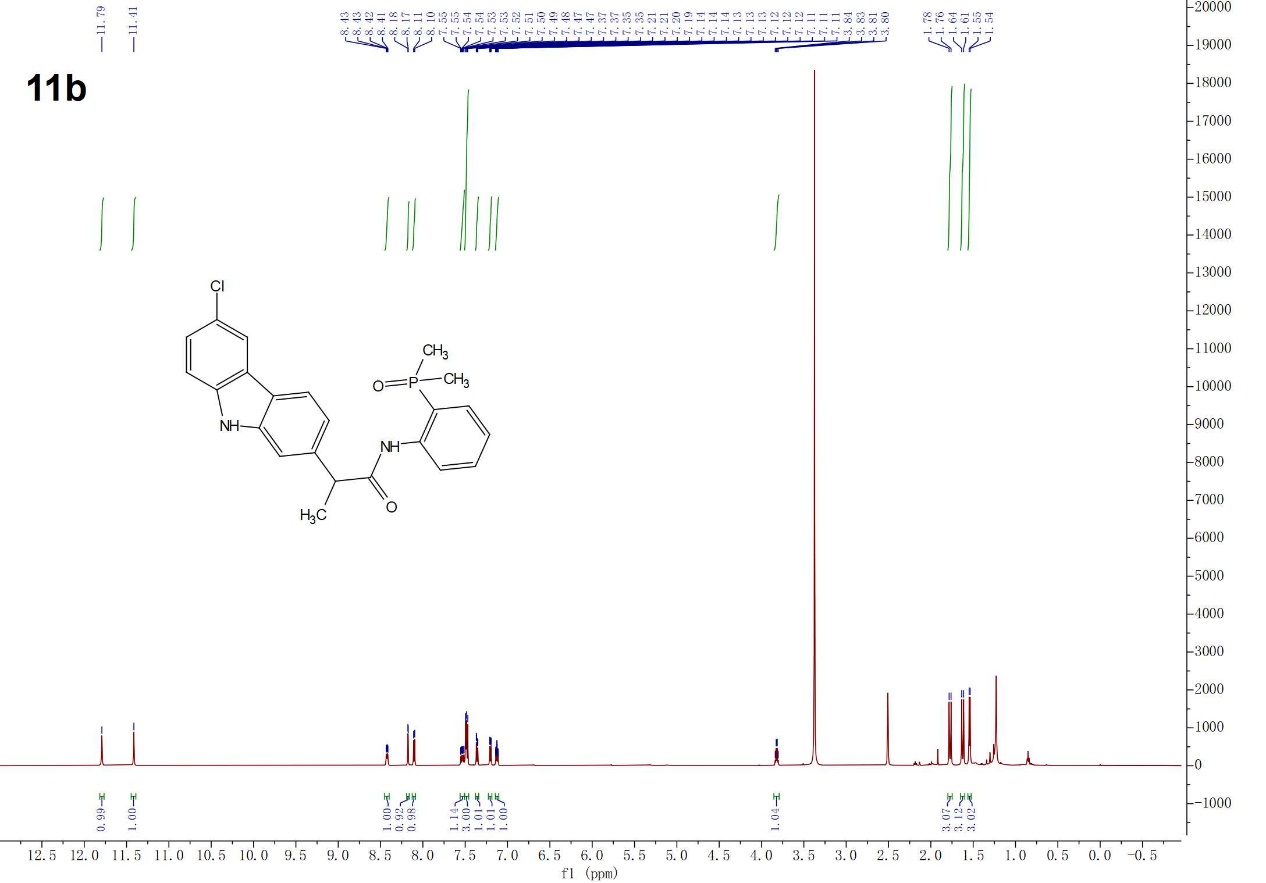

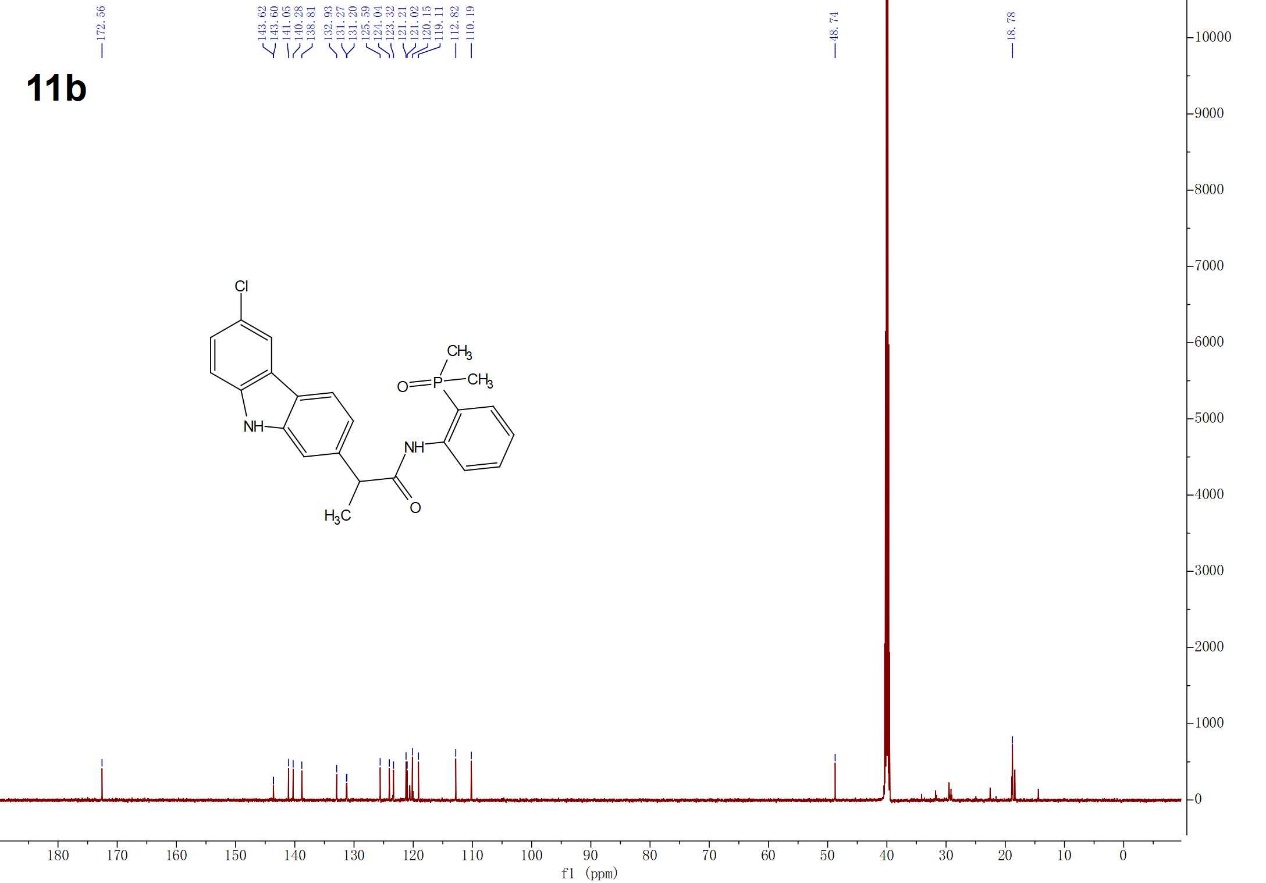


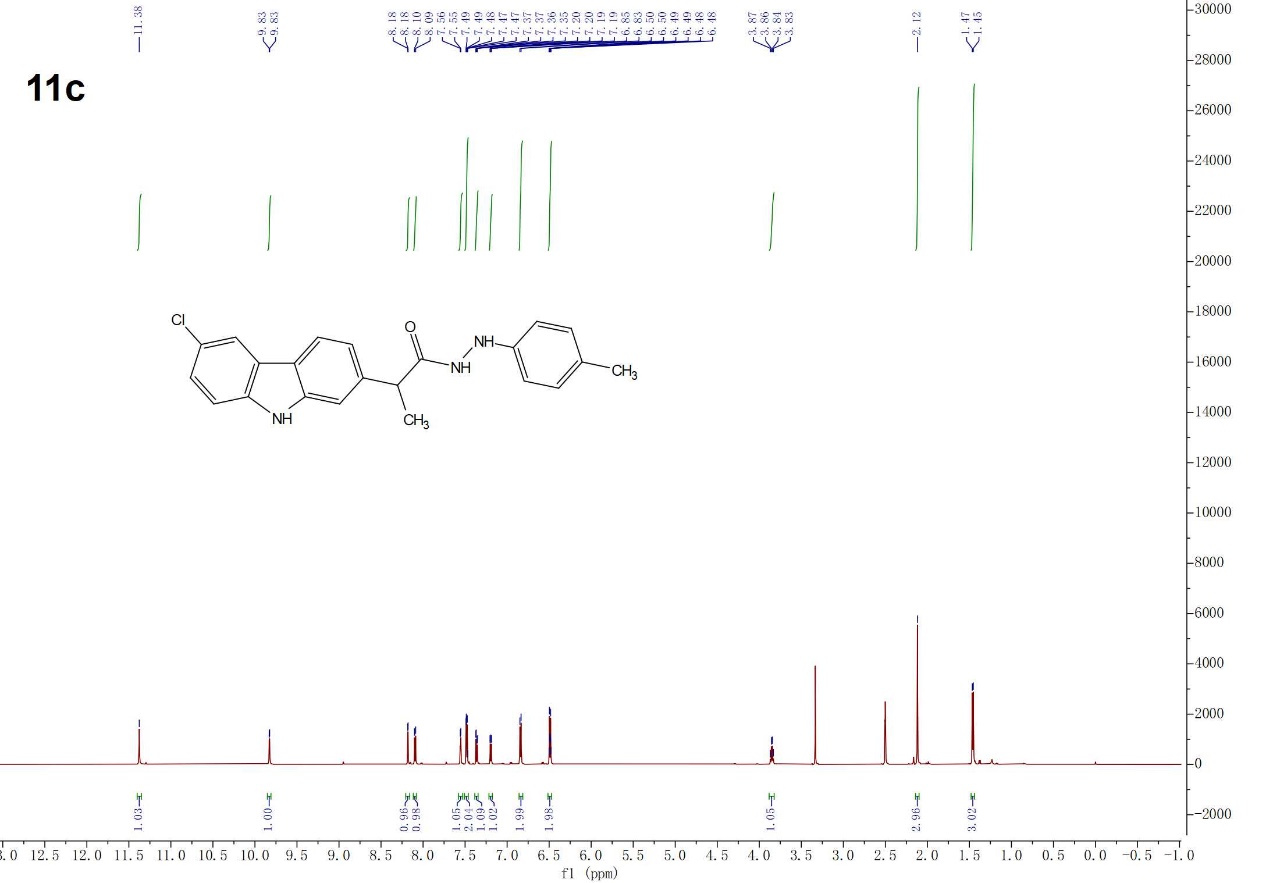

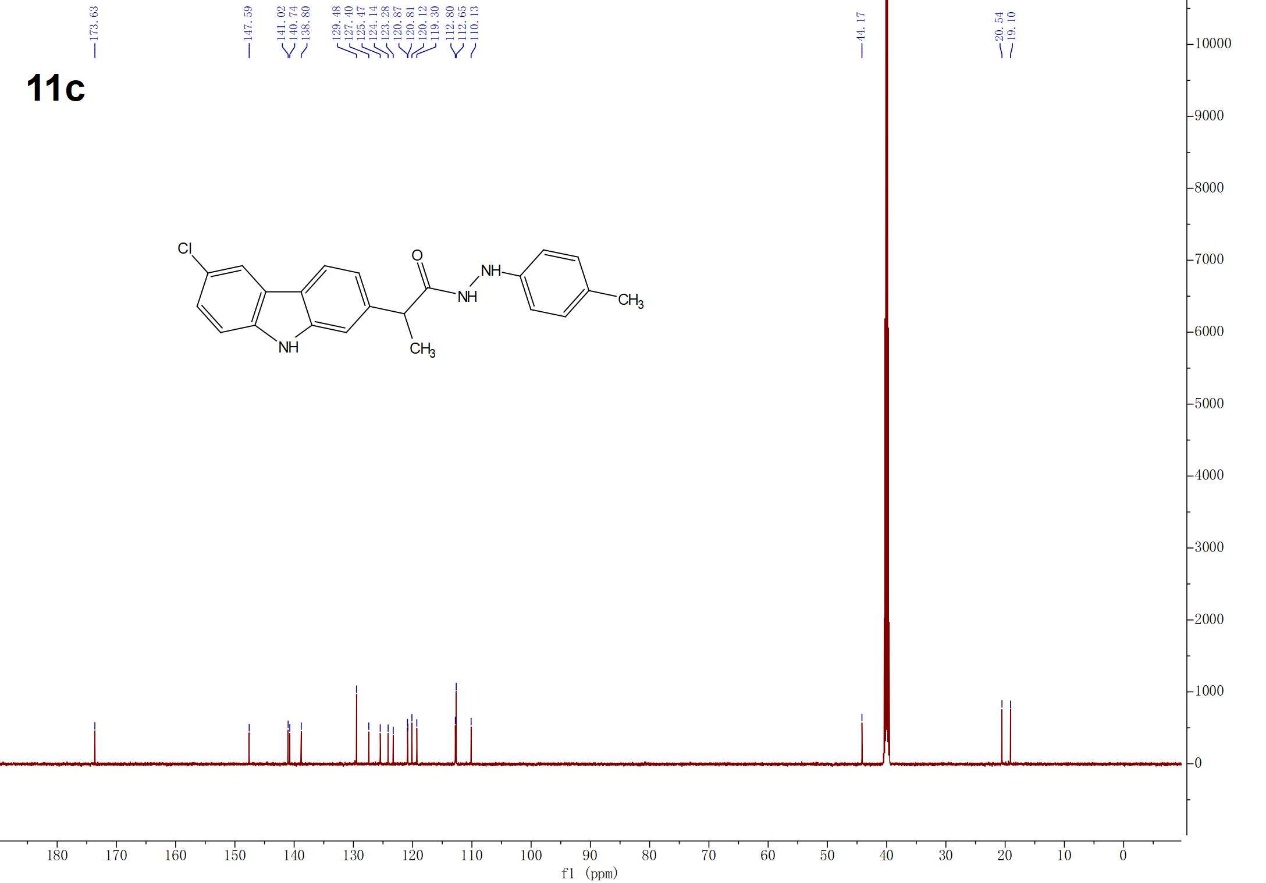


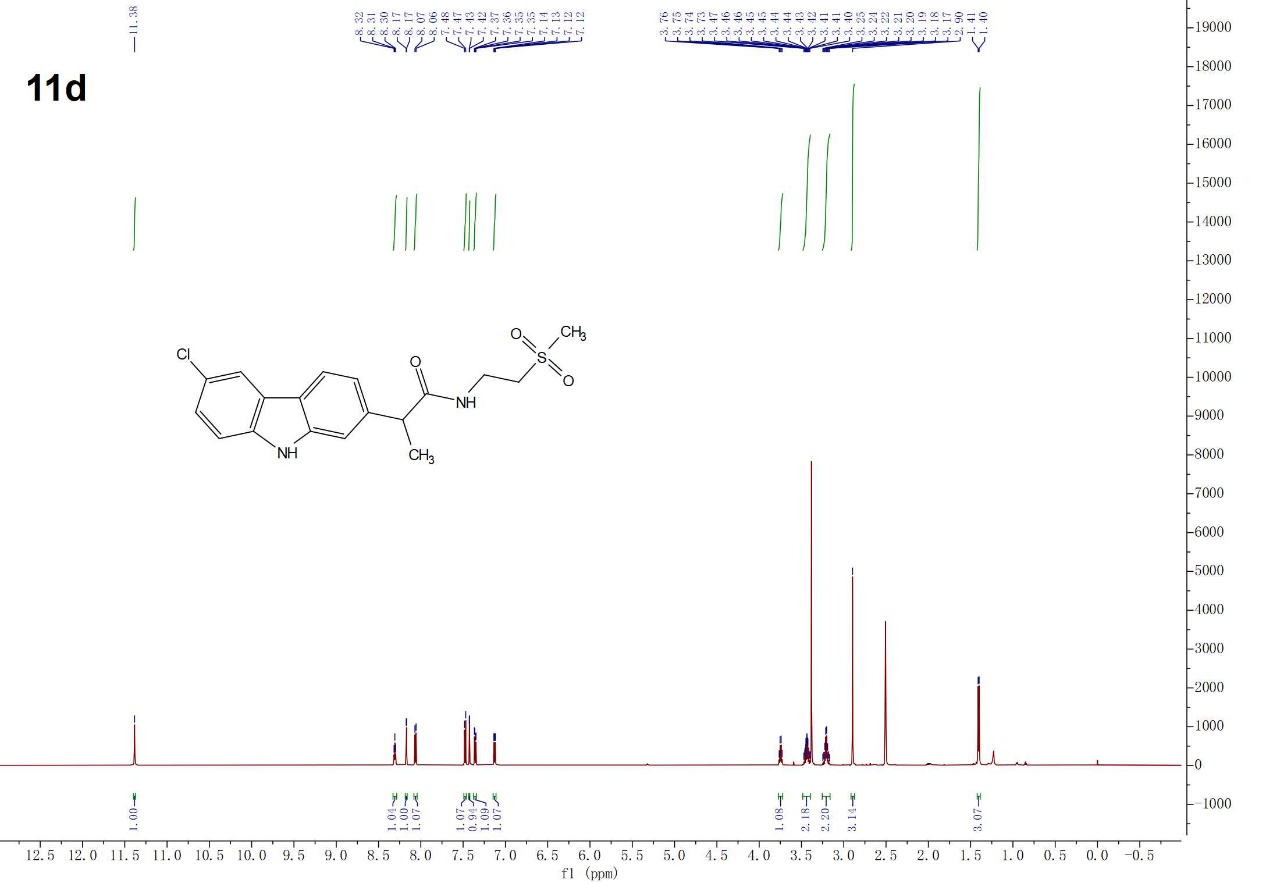

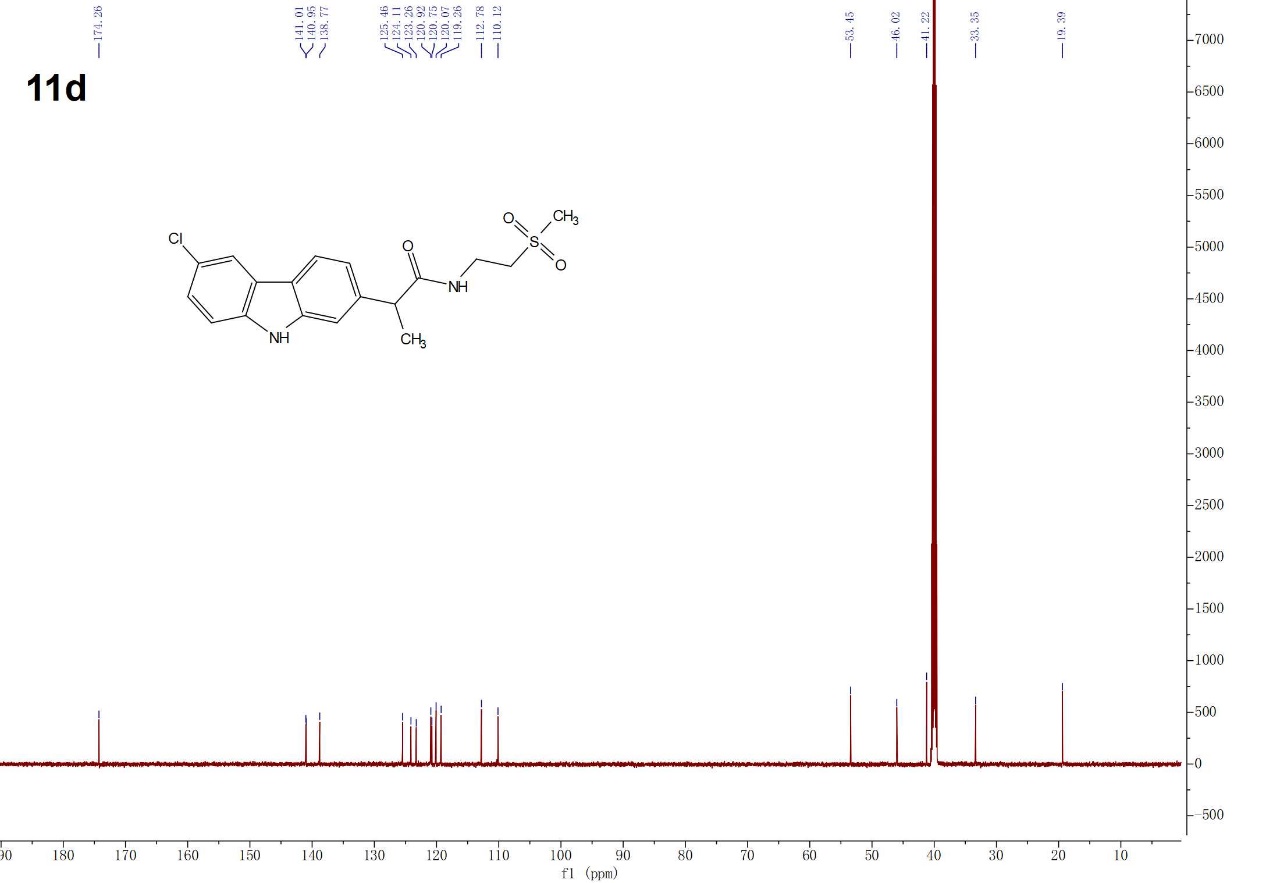


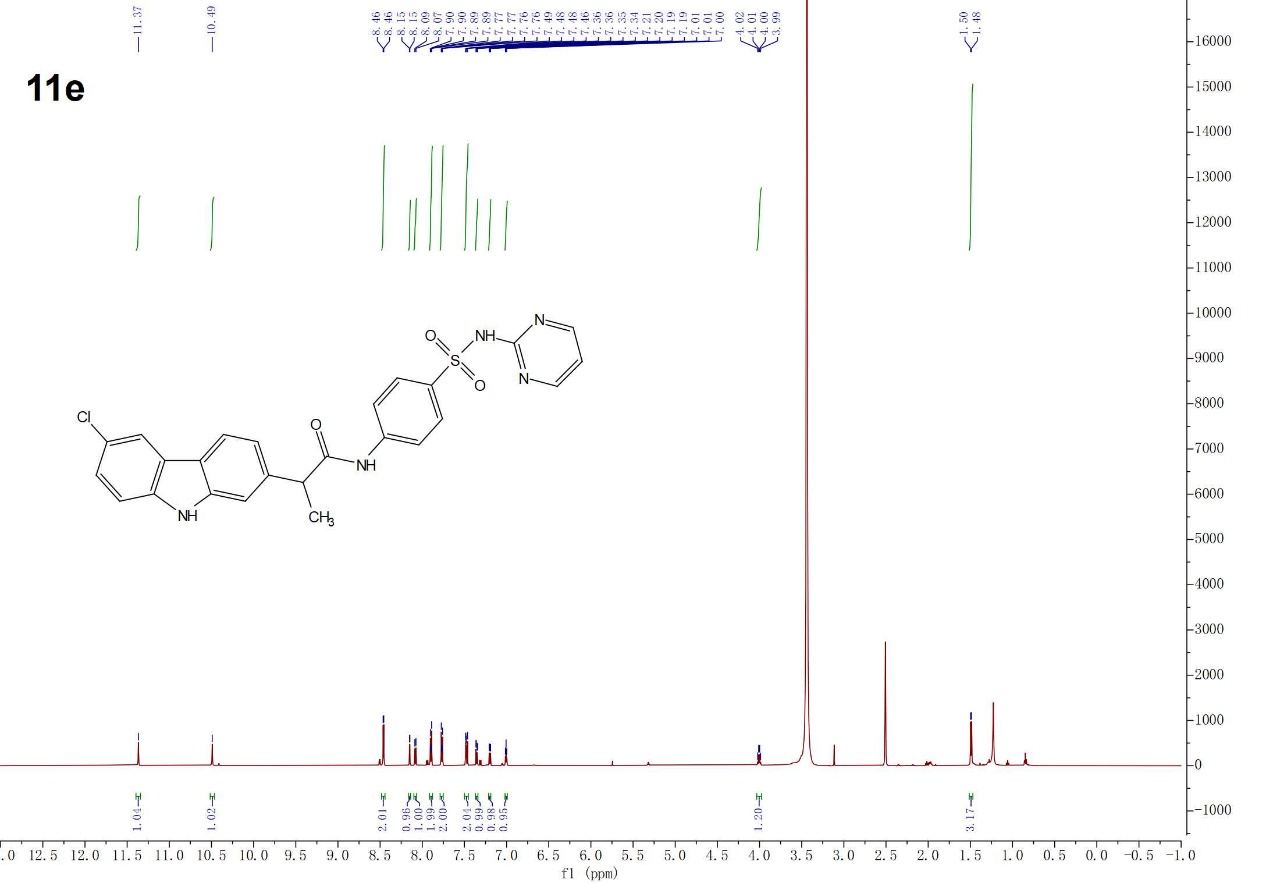


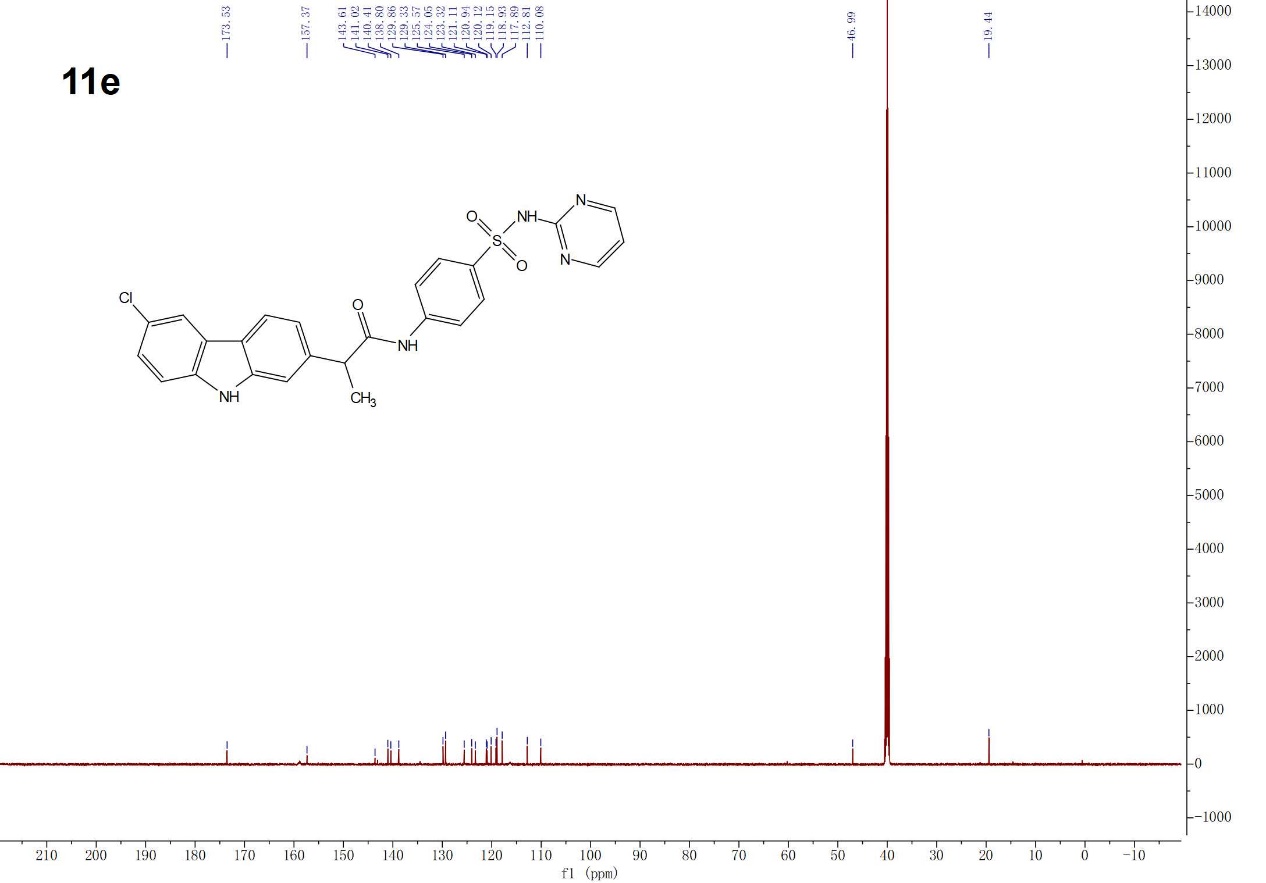


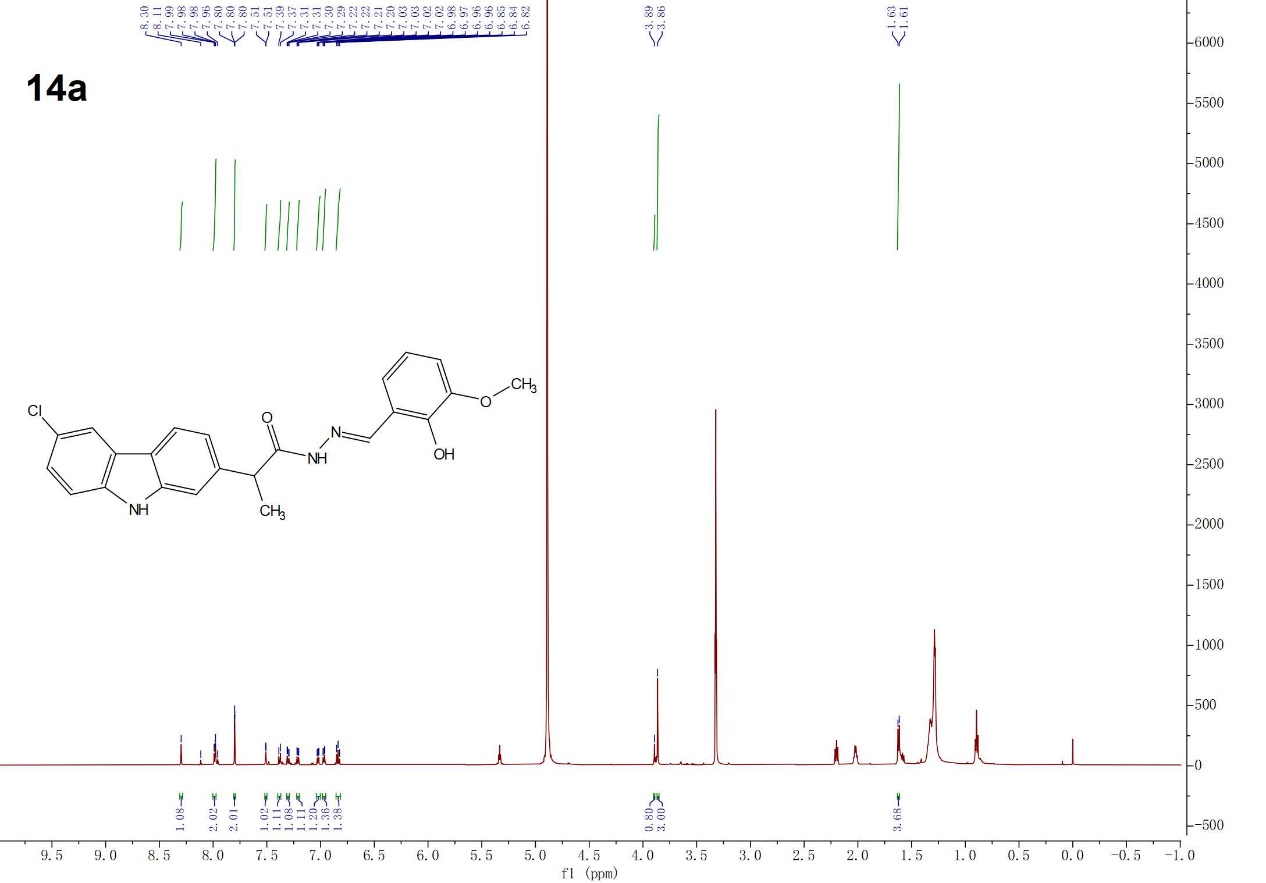


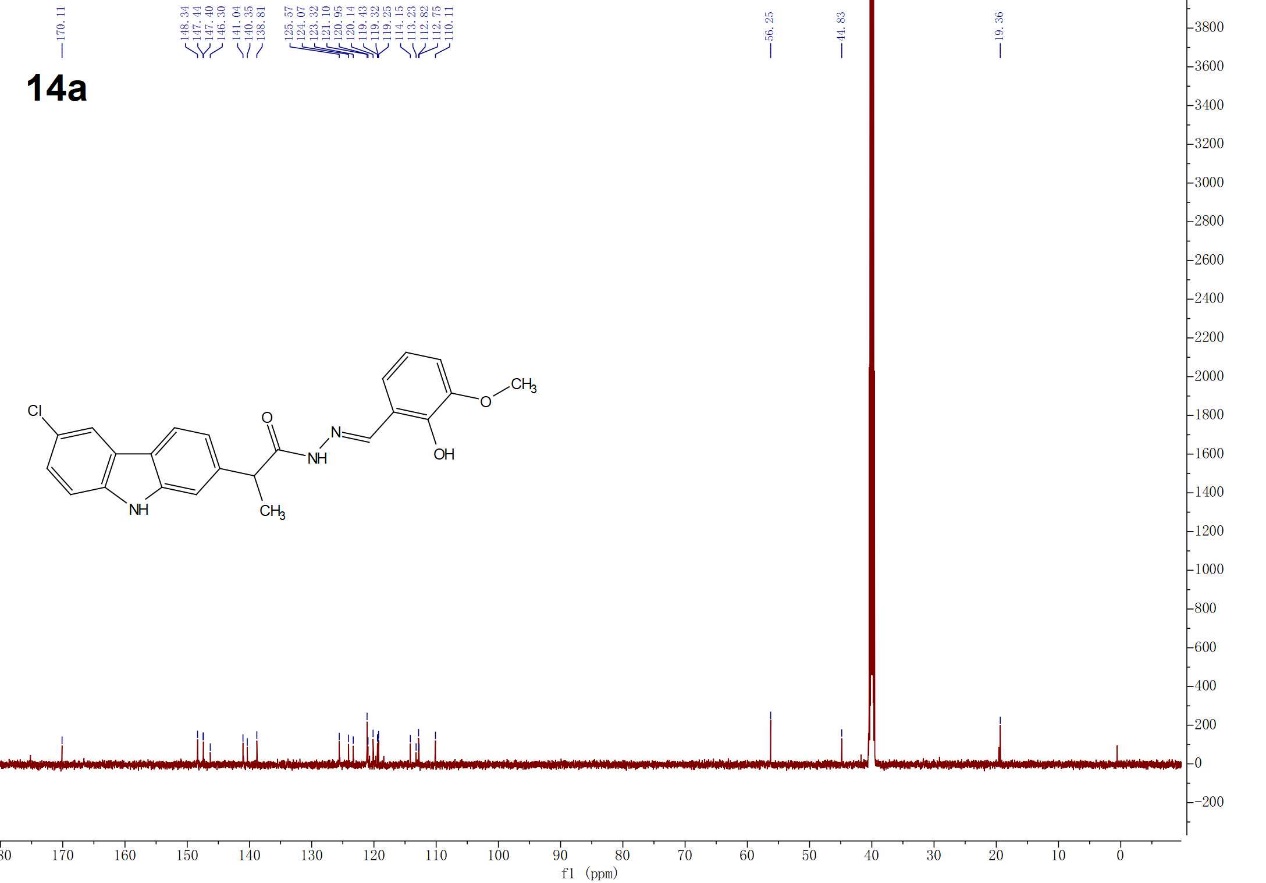


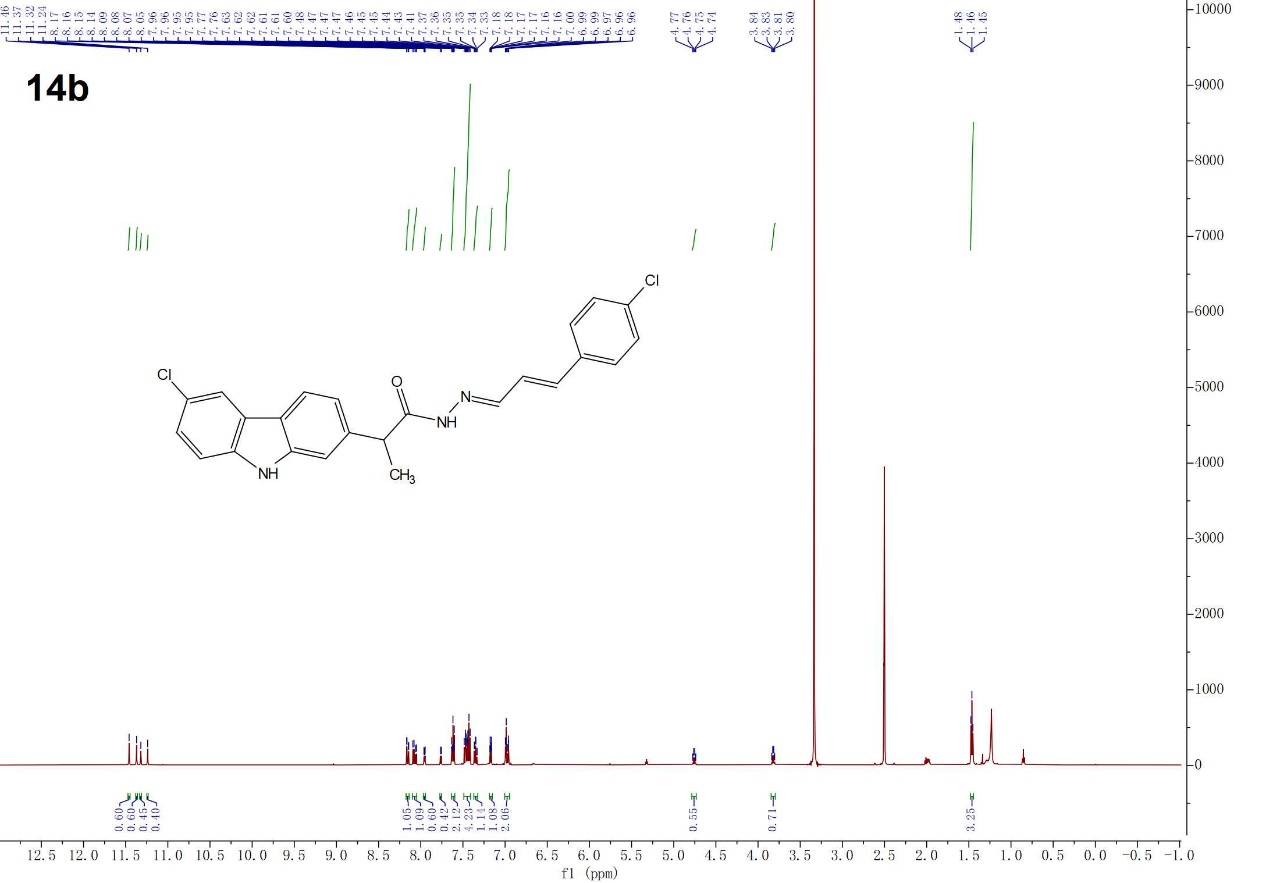

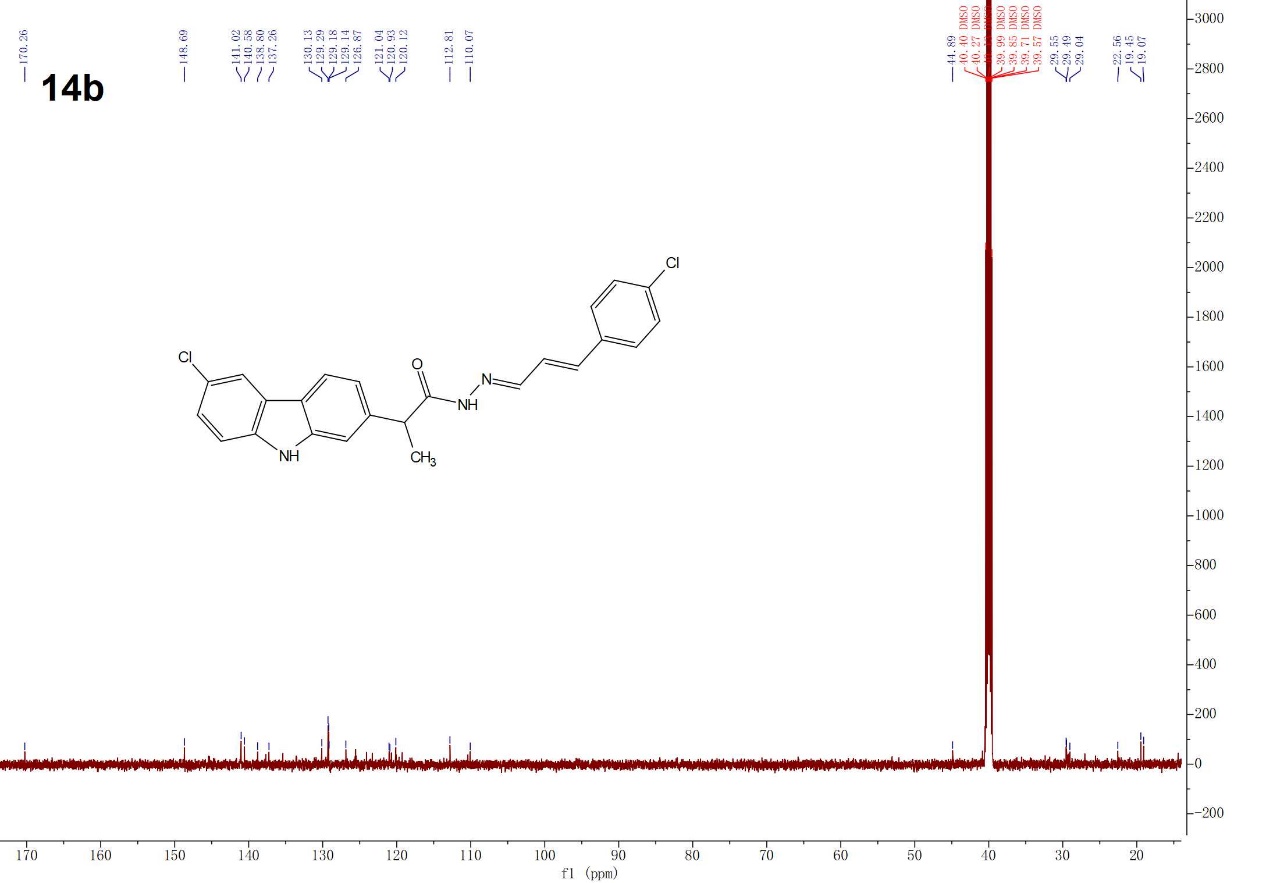


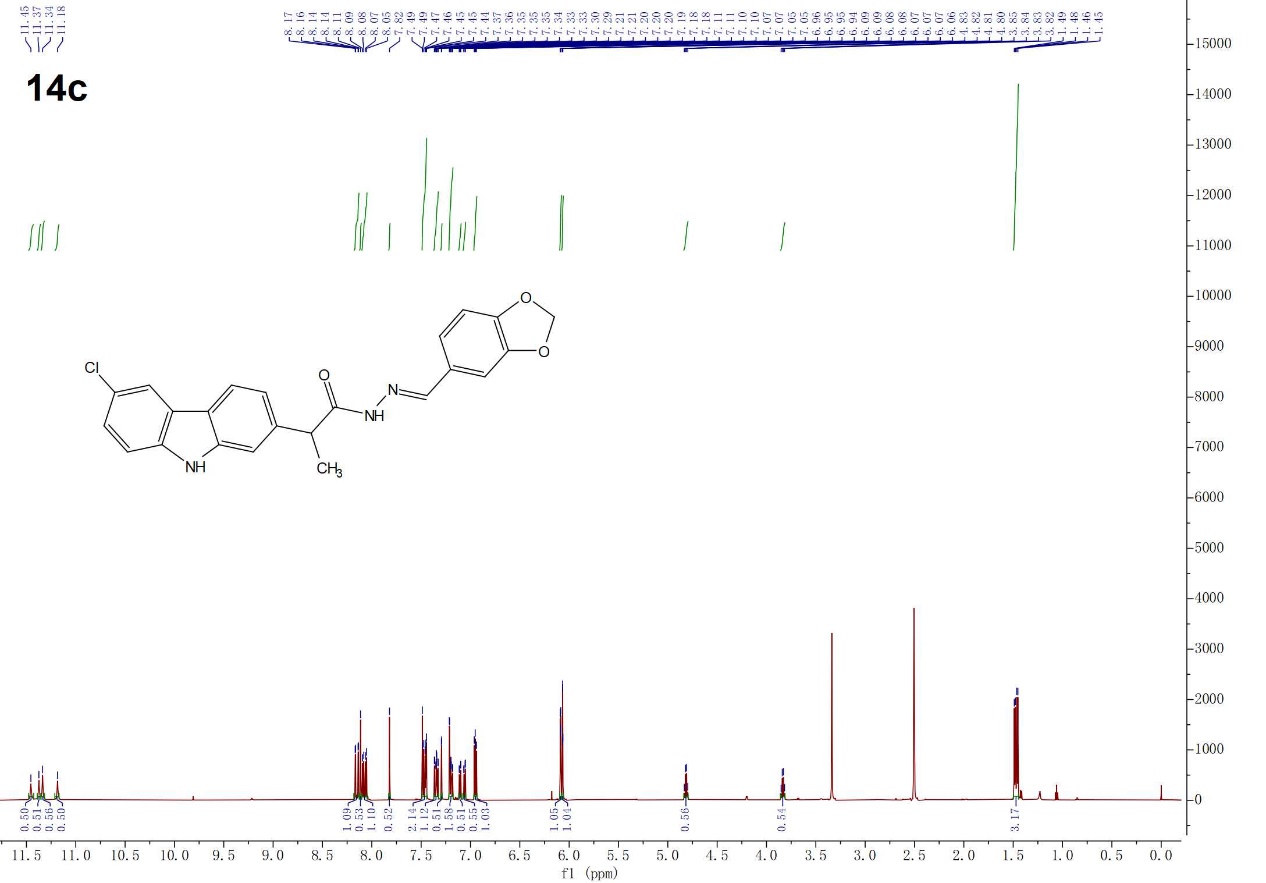

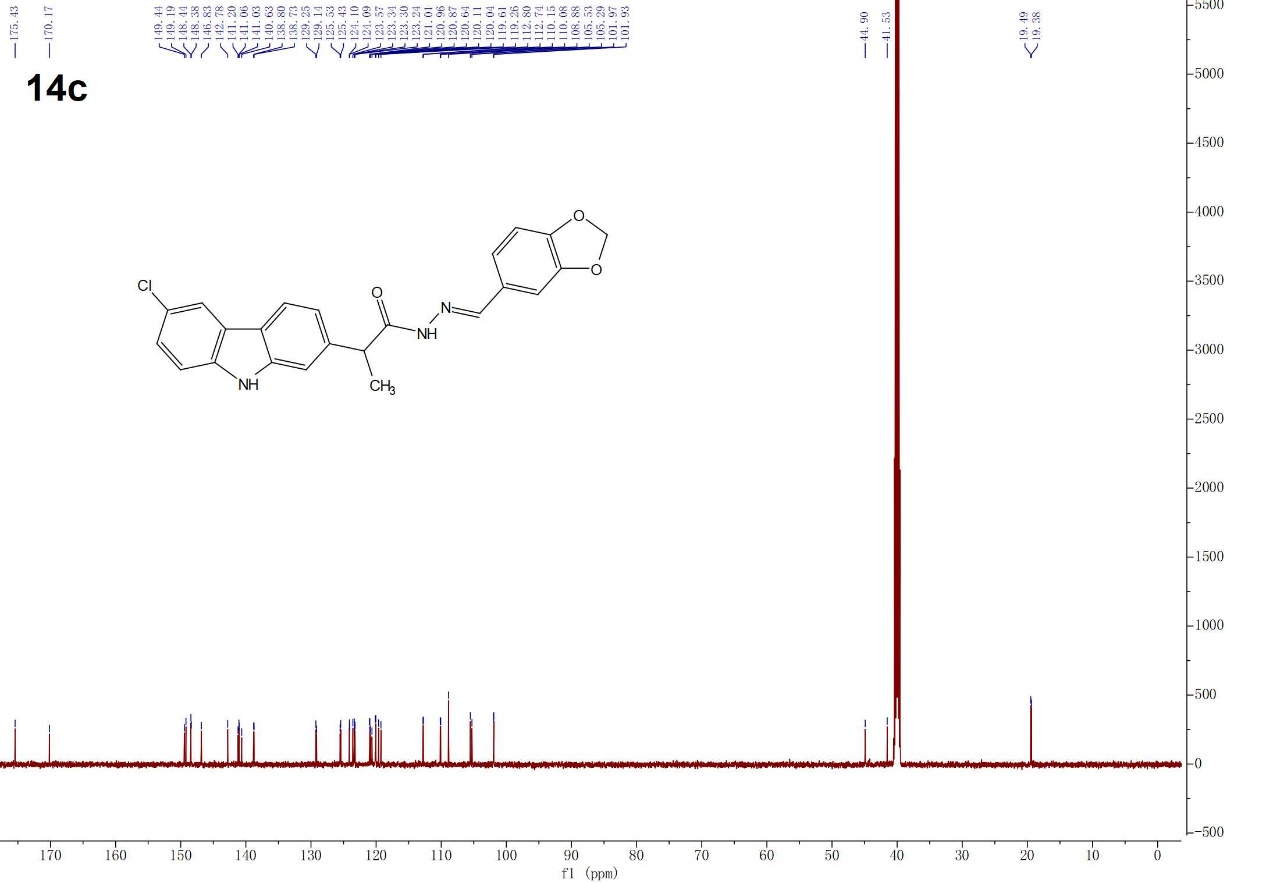


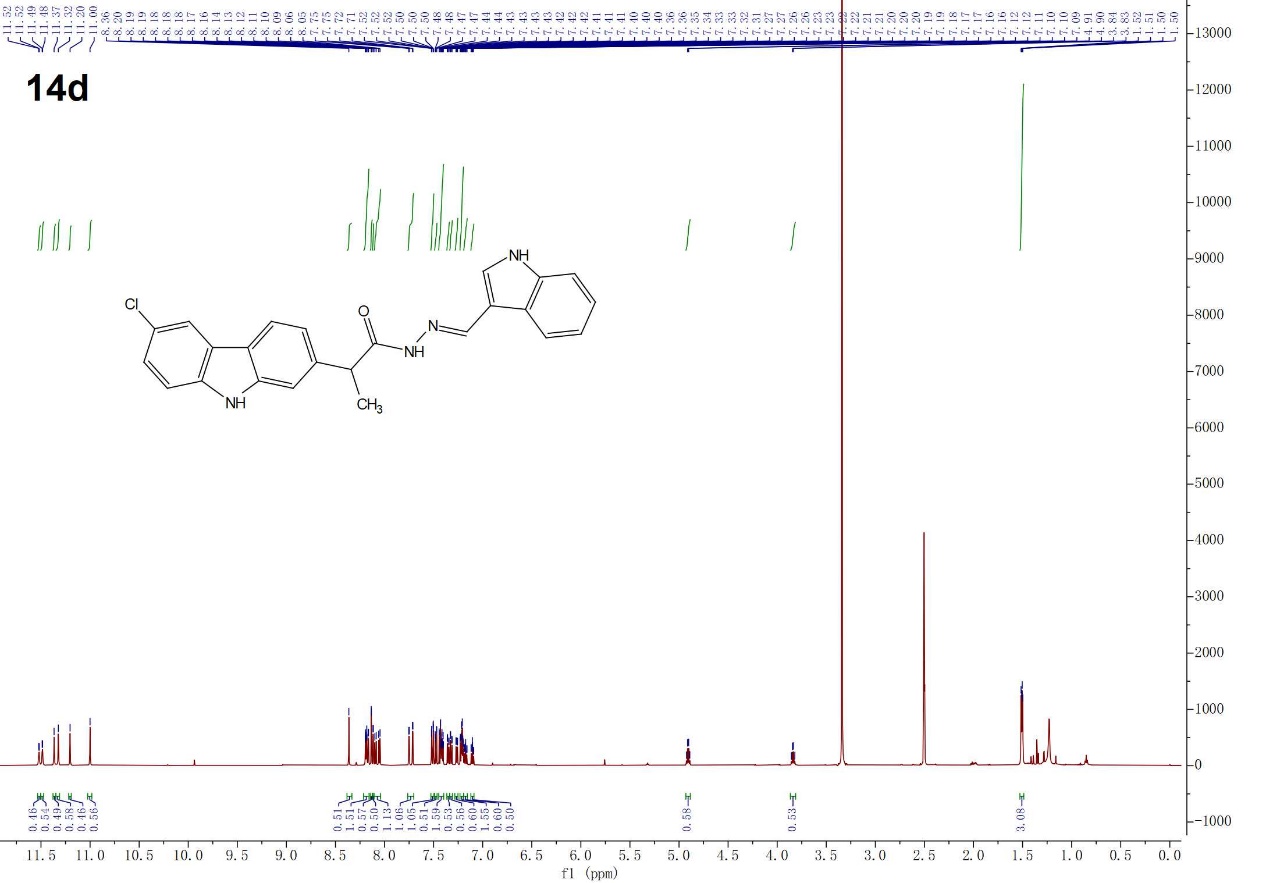

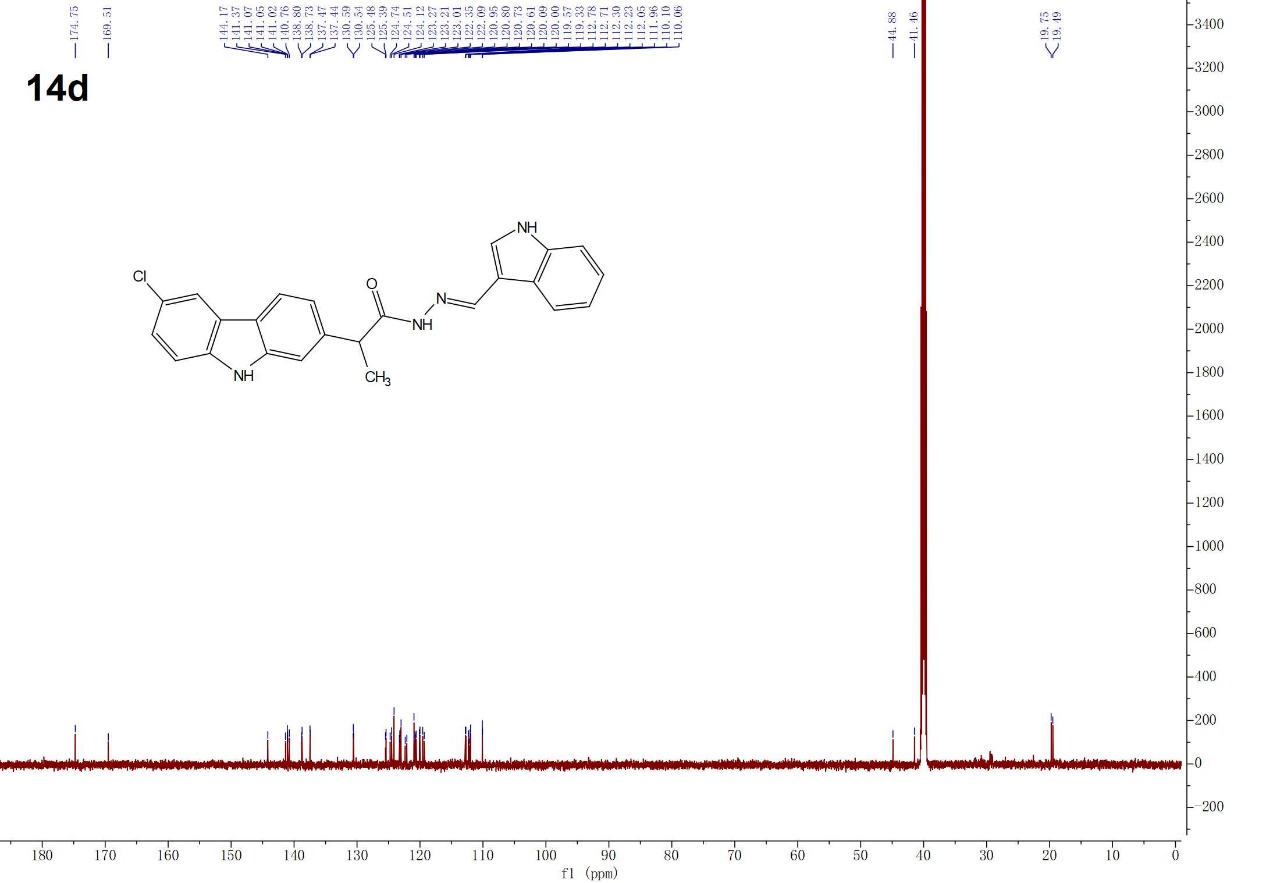


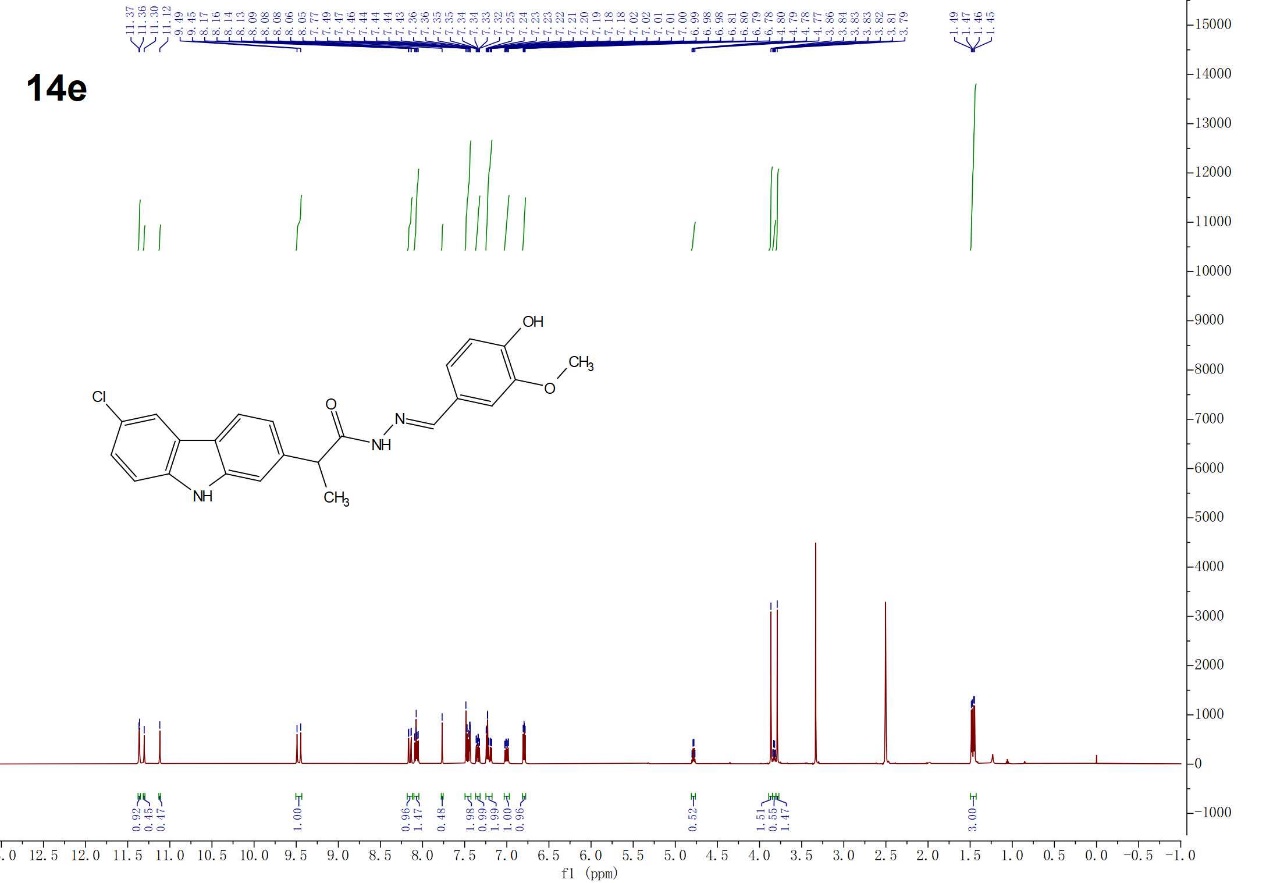

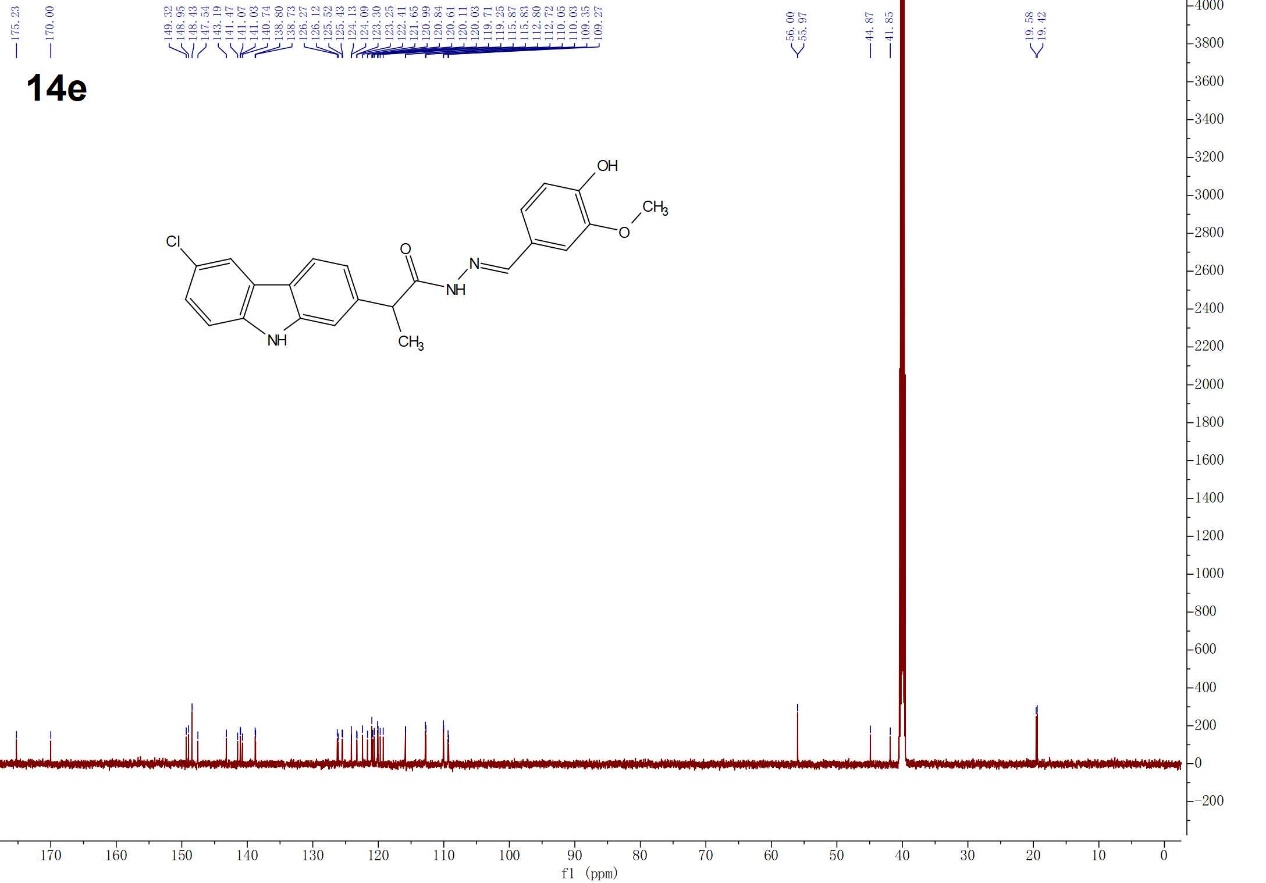


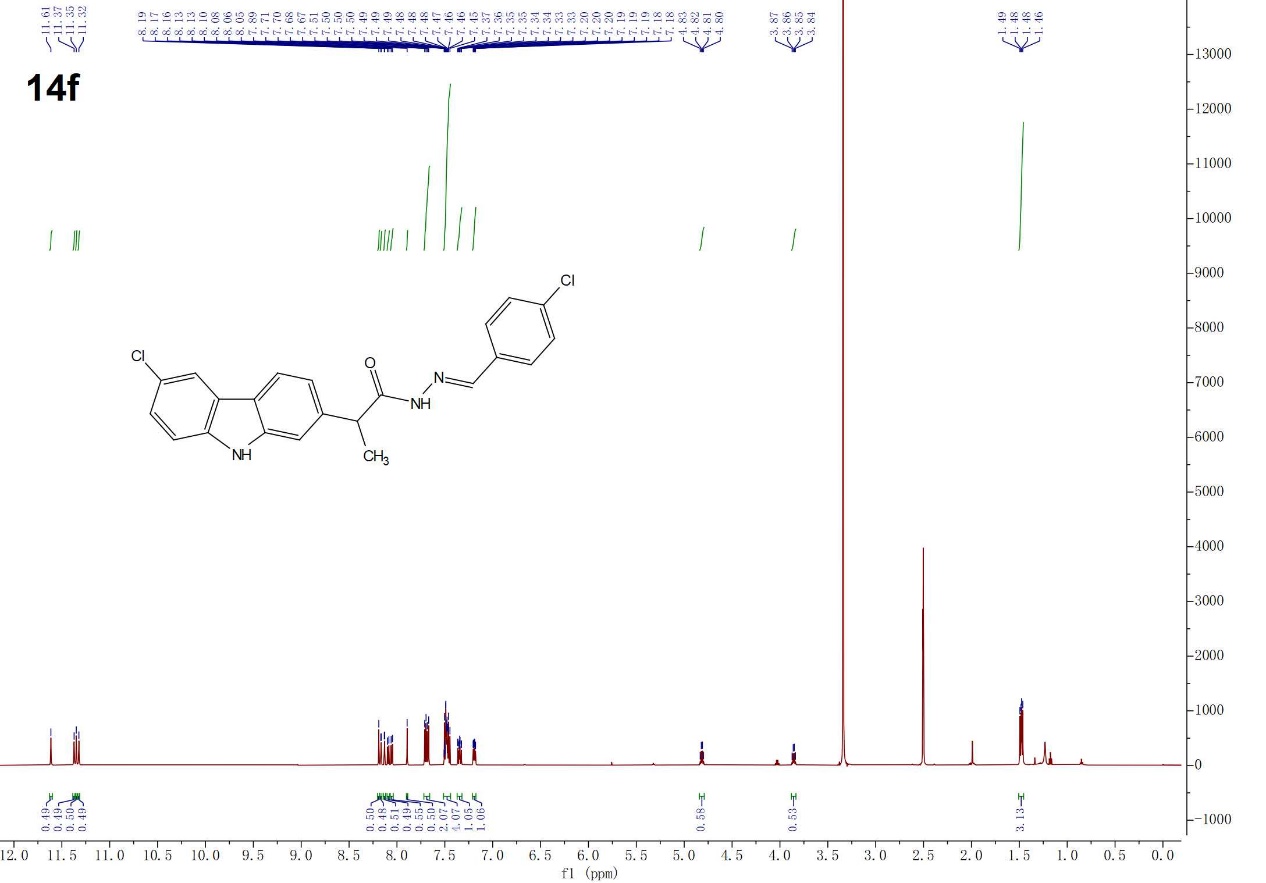

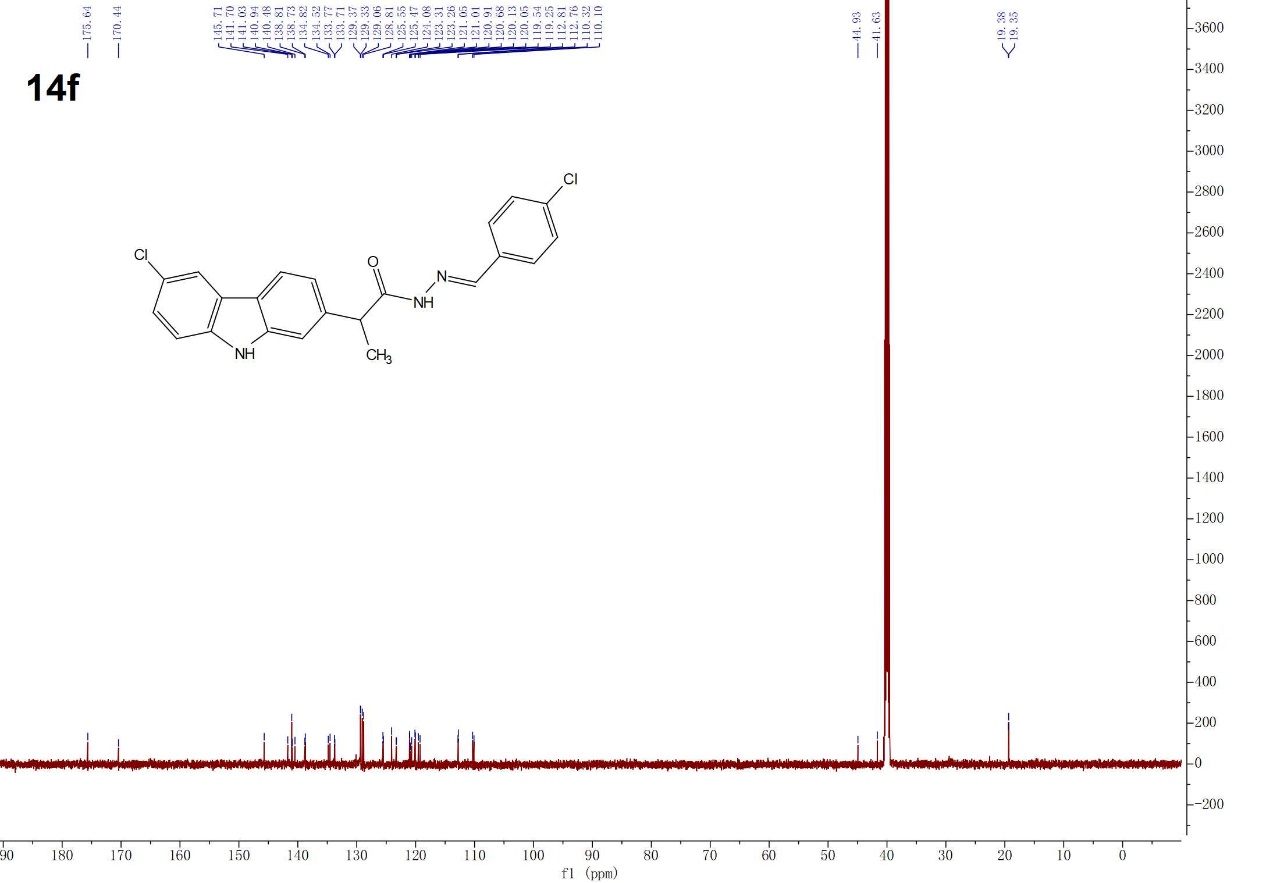


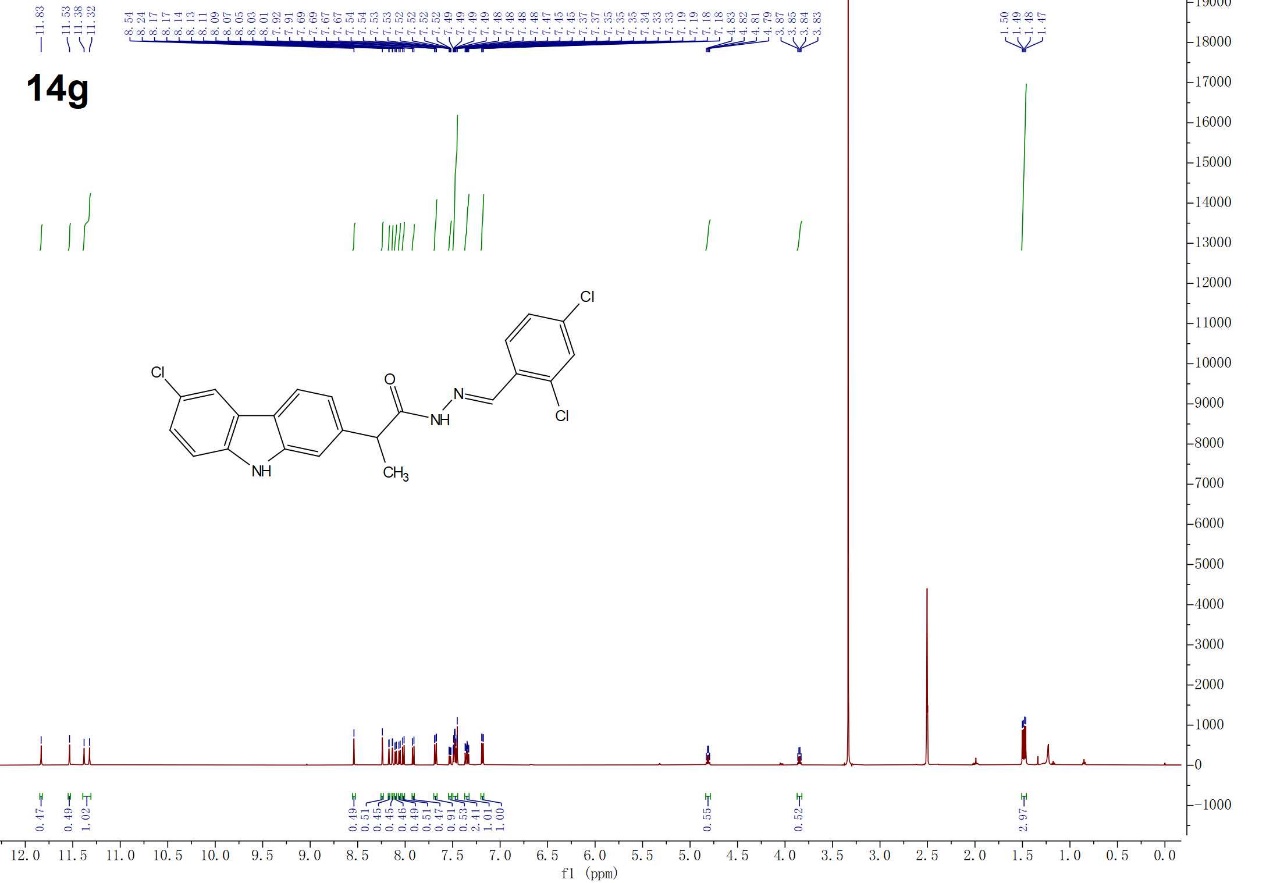

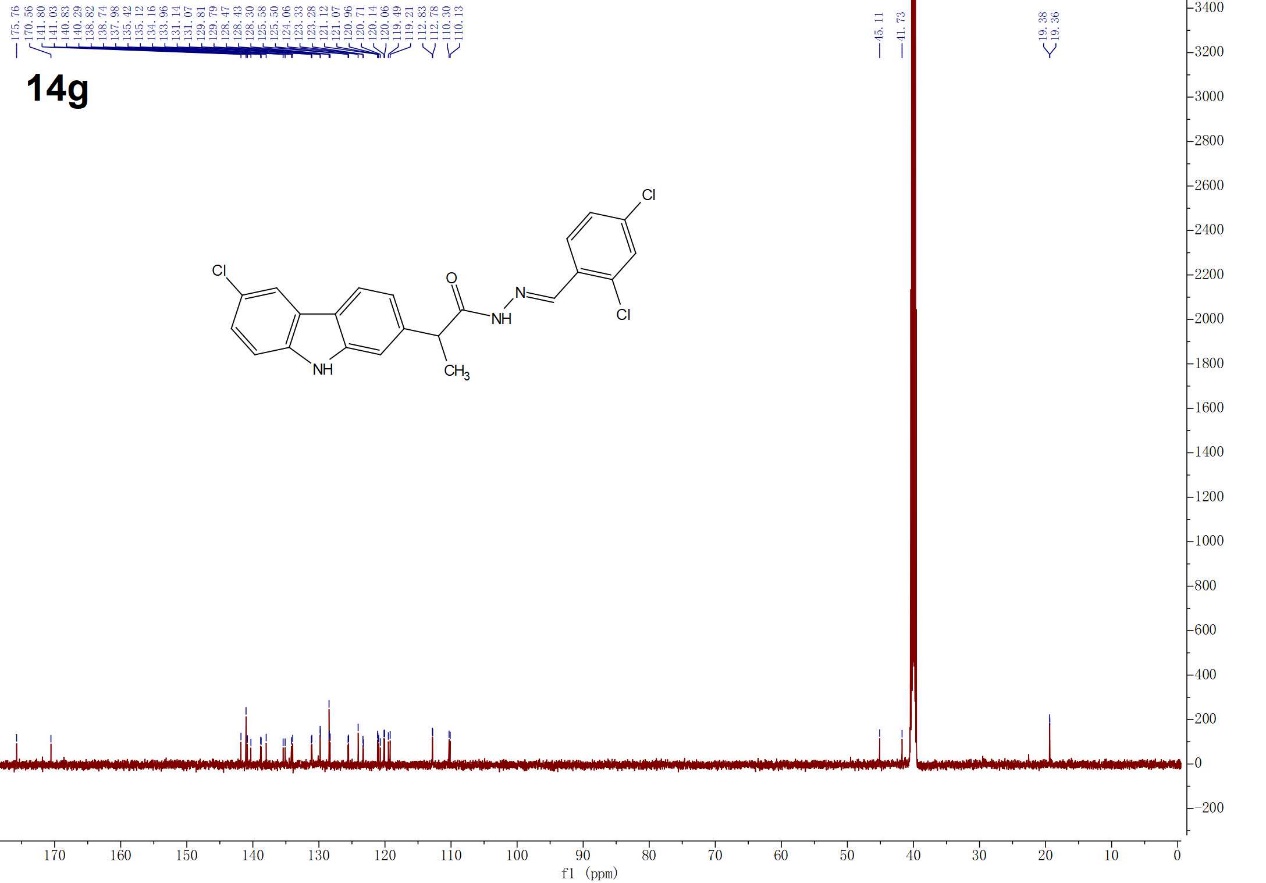

Supplement: Supplementary file 1 [file DataSheet1.docx]
